# Supplementary material for: Retention of ERK in the cytoplasm mediates the pluripotency of embryonic stem cells
Source: Stem Cell Reports. 2022 Dec 22;18(1):305–18. doi: 10.1016/j.stemcr.2022.11.017 (PMC9860118; doi:10.1016/j.stemcr.2022.11.017)
Supplement: Document S1. Figures S1–S7 [file mmc1.pdf]

Stem Cell Reports, Volume *18*

Supplemental Information

Retention of ERK in the cytoplasm mediates the pluripotency of embryonic stem cells

Avital Hacohen Lev-Ran and Rony Seger

Retention of ERK in the cytoplasm mediates the pluripotency of embryonic stem cells

Supplementary Figures

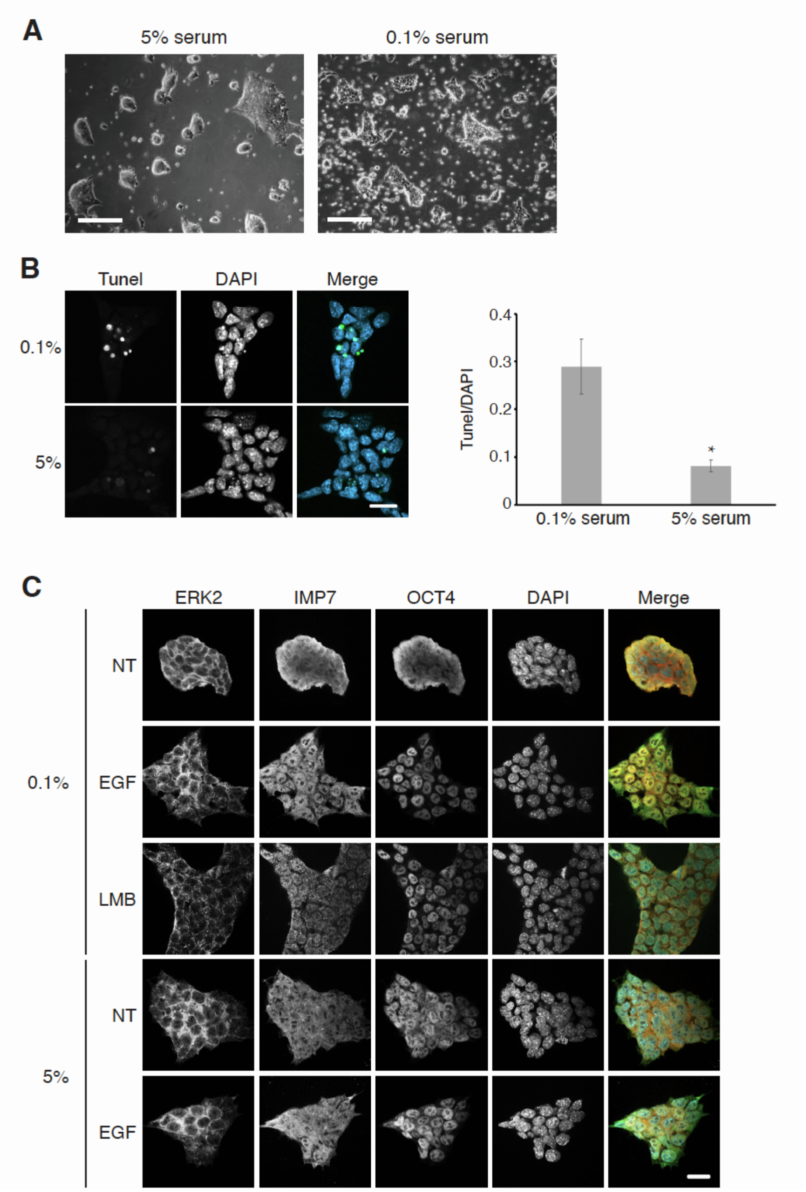

Figure S1. The effect of different starvation medium on mESC, related to Experimental Procedures. (A) Morphological studies. E14Tg2a cells were grown in two different low serum concentrations: 0.1% and 5% for 16 hr and the following parameters were followed. The morphology of colonies was visualized by a regular light microscope. Scale bars - 500μm. (**B**) TUNEL assay. The fluorescence was visualized by spinning disk confocal microscopy. Scale bar – 20 μm. The data presented in the bar-graph in the right side represents means ± SE of 3 experiments. *$P < 0.05$ as calculated by T test. (**C**) Effect of 0.1% serum on stimulated ERK translocation compared to cells starved in 5% FCS. The serum-starved (either 0.1% or 5%) cells were treated with EGF (50 ng/ml, 15 min), or left untreated, (NT). The cells starved in 0.1% FCS were also stimulated with LMB (5 ng/ml, 1 hr). Then, the cells were fixed and stained with the indicated Abs. The nuclei were detected using DAPI. Fluorescence was visualized by spinning disk confocal microscopy. Scale bar – 20 μm.

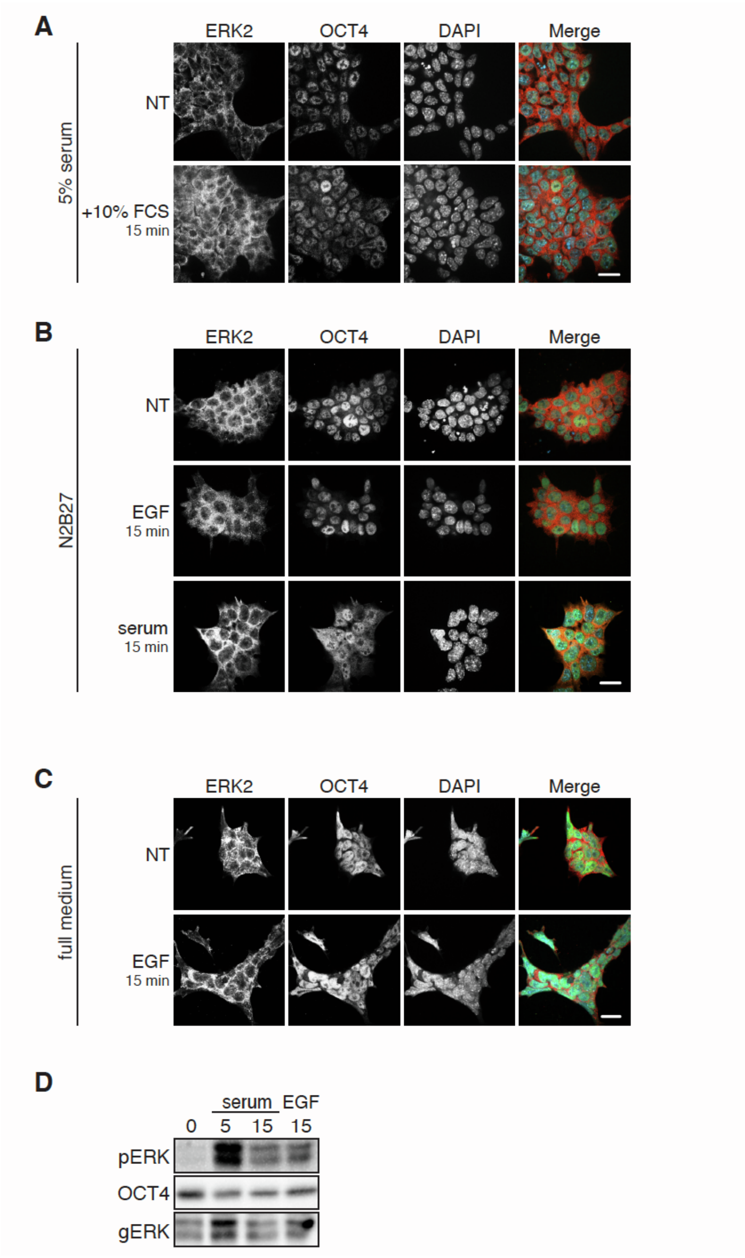

Figure S2. The effect of serum/ serum free medium on ERK translocation in mESC, related to Fig. 1 and Experimental Procedures). (**A**) Lack of nuclear ERK translocation upon serum stimulation. Fluorescence micrographs of ERK2 and OCT4 in E14Tg2a cells grown in reduced serum concentrations (5%, 16 hr) and stimulated with 10% FCS (total 15% serum, 15 min) or left untreated. Scale bar - 20 µm in all. (**B**) Lack of stimulated nuclear translocation of ERK grown in serum-free medium. Fluorescence micrographs of ERK2 and OCT4 in E14Tg2a cells grown in N2B27 (2 days), and then stimulated with EGF (50 ng/ml, 15 min), FCS (15% serum, 15 min) or left untreated as control (NT). (**C**) Lack of stimulated ERK translocation without starvation. Fluorescence micrographs of ERK2 and OCT4 in E14Tg2a cells grown in full medium (15% serum, 2 days) and stimulated with EGF (50 ng/ml, 15 min), or left untreated as control (NT). Fluorescence was visualized by spinning disk confocal microscopy. (D) ERK is activated by EGF and serum in E14Tg2a cells grown without serum. The cells were grown in N2B27 (2 days) and then were either stimulated with EGF (50 ng/ml, 15 min), FCS (15% serum, 5 and 15 min) or left untreated (0). The cells were then harvested and subjected to Western blotting using the indicated Abs.

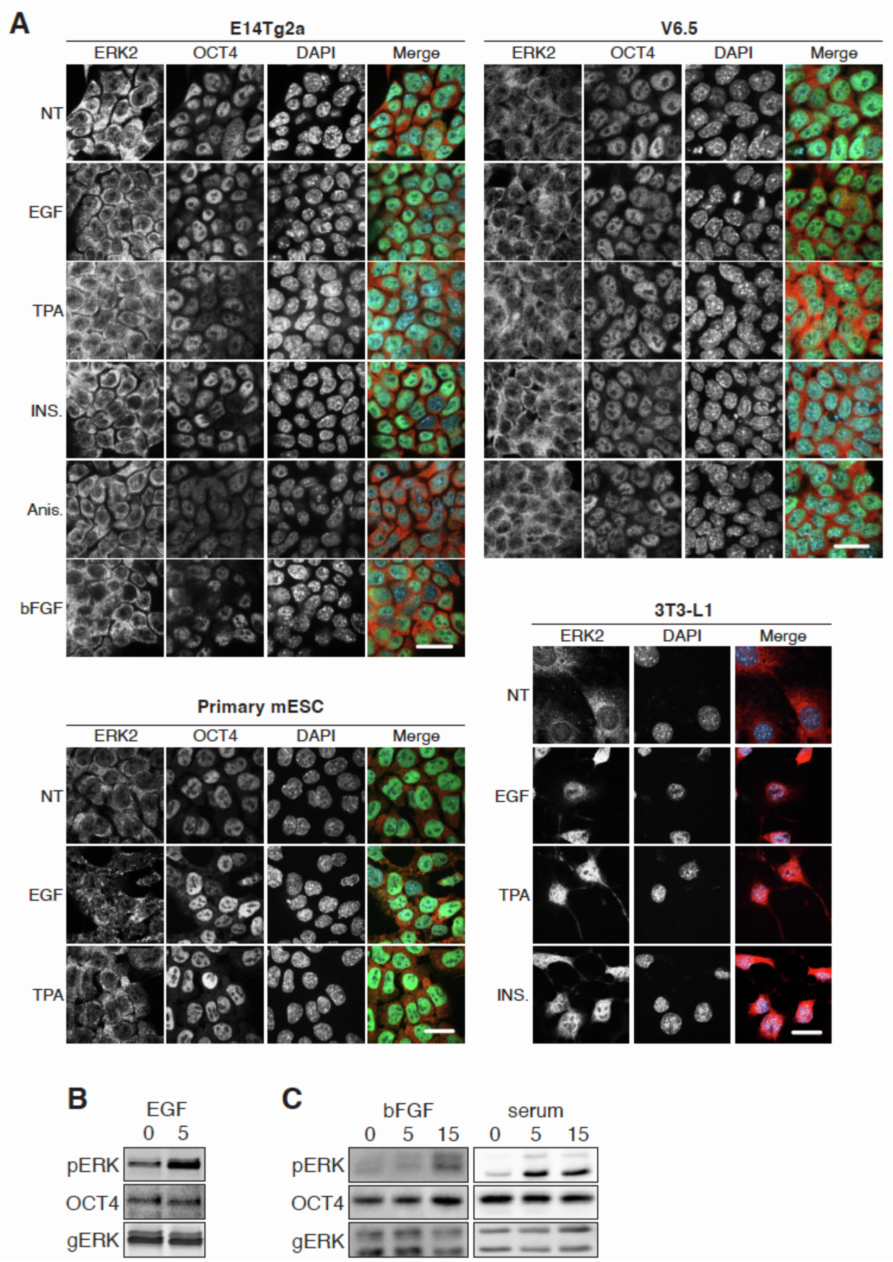

Figure S3. ERK1/2 localization in different cells and stimuli, related to Fig. 1. (**A**) Lack of nuclear translocation in various stem cells. Fluorescence micrographs of ERK2 and OCT4 in E14Tg2a, V6.5, primary (blastocyst-derived) stem cells and 3T3-L1 cells. The indicated cells were grown with reduced serum concentrations for 16 hr (mESC - 5%; 3T3-L1 – 0.1%) and then were stimulated with EGF (50 ng/ml), TPA (250 nM), insulin (100 nM; INS.), anisomycin (1 µg/ml; Anis.) and bFGF (50 ng/ml) for 15 min or left untreated (NT) as indicated. The nuclei were detected using DAPI. Fluorescence was visualized by spinning disk confocal microscopy. Scale bars - 20 µm in all. (**B**) ERK is activated by EGF in primary stem cells. The cells were grown in reduced serum concentration (5%, 16 hr) as above, and then were either stimulated with EGF (50 ng/ml, 5 min) or left untreated (0). The cells were then harvested and subjected to Western blotting using the indicated Abs. (**C**) ERK is activated by bFGF and Serum. E14Tg2a cells were grown in reduced serum concentration (5%, 16 hr) as above, and then were either stimulated with either bFGF (50 ng/ml, 5 or 15 min), 10% FCS (total 15% serum, 5 or 15 min) or left untreated (0). The cells were then harvested and subjected to Western blotting using the indicated Abs.

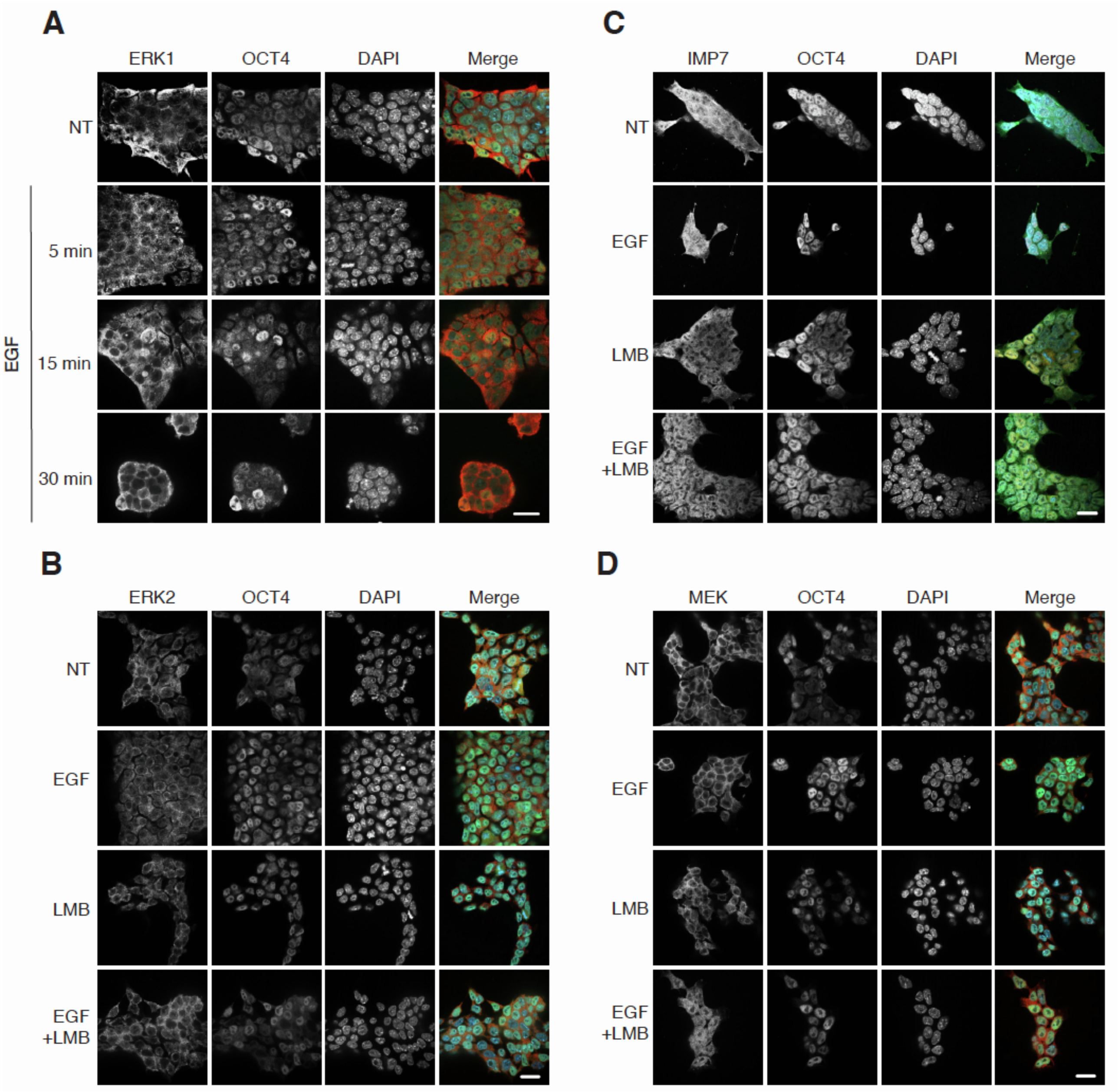

Figure S4. Subcellular localization of ERK1+2, IMP7 and MEK upon various stimulation of mESC, related to Fig. 1. (A) Subcellular localization of ERK1 upon EGF stimulation in different time points. Fluorescence micrographs of ERK1 (using specific Ab) in E14Tg2a cells grown in reduced serum concentrations (5%, 16 hr) and then either stimulation with EGF (50 ng/ml, 5, 15 and 30 min) or no stimulation (NT). Next the cells were fixed and stained with the indicated Abs. The nuclei were detected using DAPI. Fluorescence was visualized by spinning disk confocal microscopy. Scale bars - 20 µm in all. (**B-D**) Leptomycin B does not affect ERK2 in mESC. E14Tg2a cells were grown in low serum concentrations (5%, 16 hr) and then treated with EGF (50 ng/ml, 15 min), Leptomycin B (LMB, 5 ng/ml, 1 hr), LMB together with EGF that was added for the last 15 min of LMB treatment (total 1 hr) or left untreated as control (NT). Then the cells were fixed and stained with the ERK (B), IMP7 (C) or MEK (D) Abs with anti OCT4 Ab and DAPI. Fluorescence was visualized by spinning disk confocal microscopy.

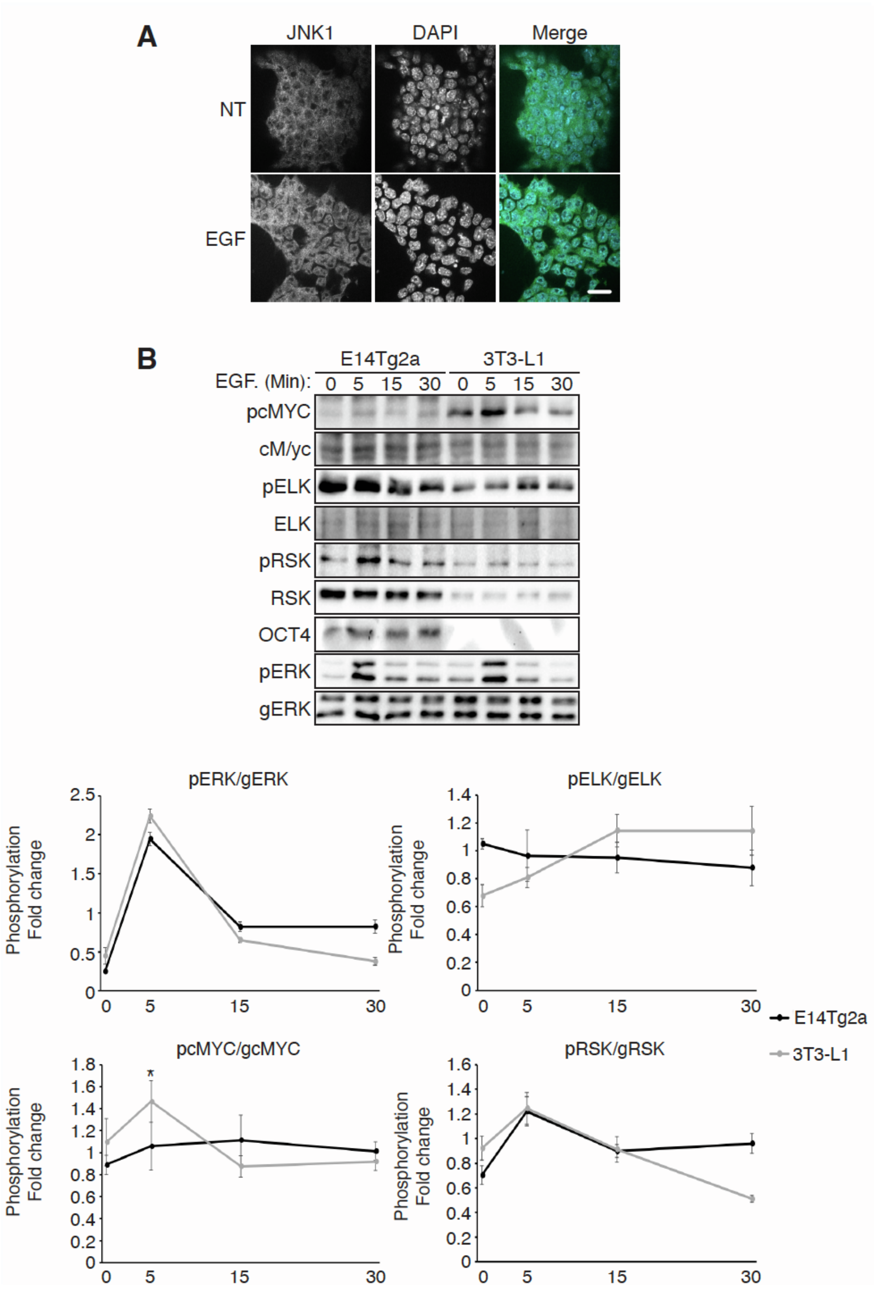

Figure S5: Studies on JNK1 and ERK substrates localization or activation upon EGF treatment, related to Fig. 2. (A) Nuclear translocation of JNK1 upon EGF stimulation of mESC. E14Tg2a cells were grown in low serum concentrations (5%, 16 hr) and then treated with EGF (50 ng/ml, 15 min), or left untreated as control (NT). Then the cells were fixed and stained with anti JNK1 Ab and DAPI. Fluorescence was visualized by spinning disk confocal microscopy. Scale bar - 20 µm. **(B)** Phosphorylation of cytoplasmic but not nuclear ERK1/2 substrates. E14Tg2a and 3T3-L1 cells grown in reduced serum concentration (E14Tg2 - 5%; 3T3-L1 – 0.1%) for 16 hr and either stimulated (E14Tg2a cells with EGF (50 ng/ml) and 3T3-L1 with insulin (100 nM)) for 5, 15 or 30 min or left untreated (0). The cells were then harvested and subjected to western blot analysis with the indicated Abs. The quantification in the graph below represent means ± SE of three experiments. **P* <0.05 by Tukey test, indicate significant changes between stem cells line (E14Tg2a) and the control cells (3T3-L1).

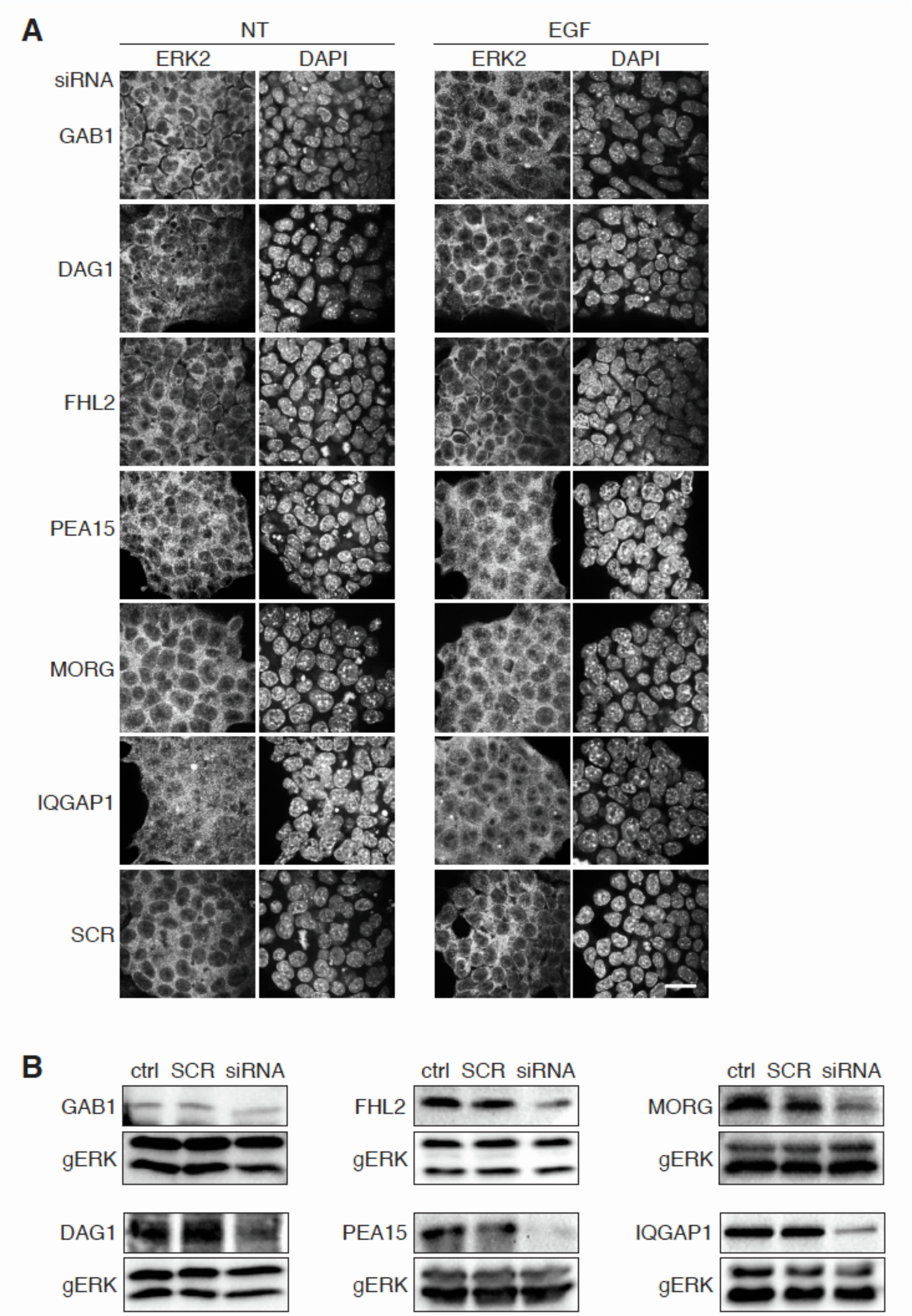

Figure S6: The effect of reduced expression of anchoring proteins on ERK1/2 localization, related to Fig. 3. (A) ERK localization. E14Tg2a cells were treated with 100nM of siRNA against GAB1, DAG1, FHL2, PEA15, MORG, IQGAP1 or scrambled (SCR) siRNA, then grown in low serum concentrations (5%, 16 hr) and the either stimulated with EGF (50 ng/ml, 15 min) or left untreated (NT). Next, the cells were fixed and stained with the indicated Abs. The fluorescence was visualized by spinning disk confocal microscopy. Scale bar - 20 µm. **(B)** Western blot analysis. The cells were treated as above, followed by extraction and Western blotting with the indicated Abs.

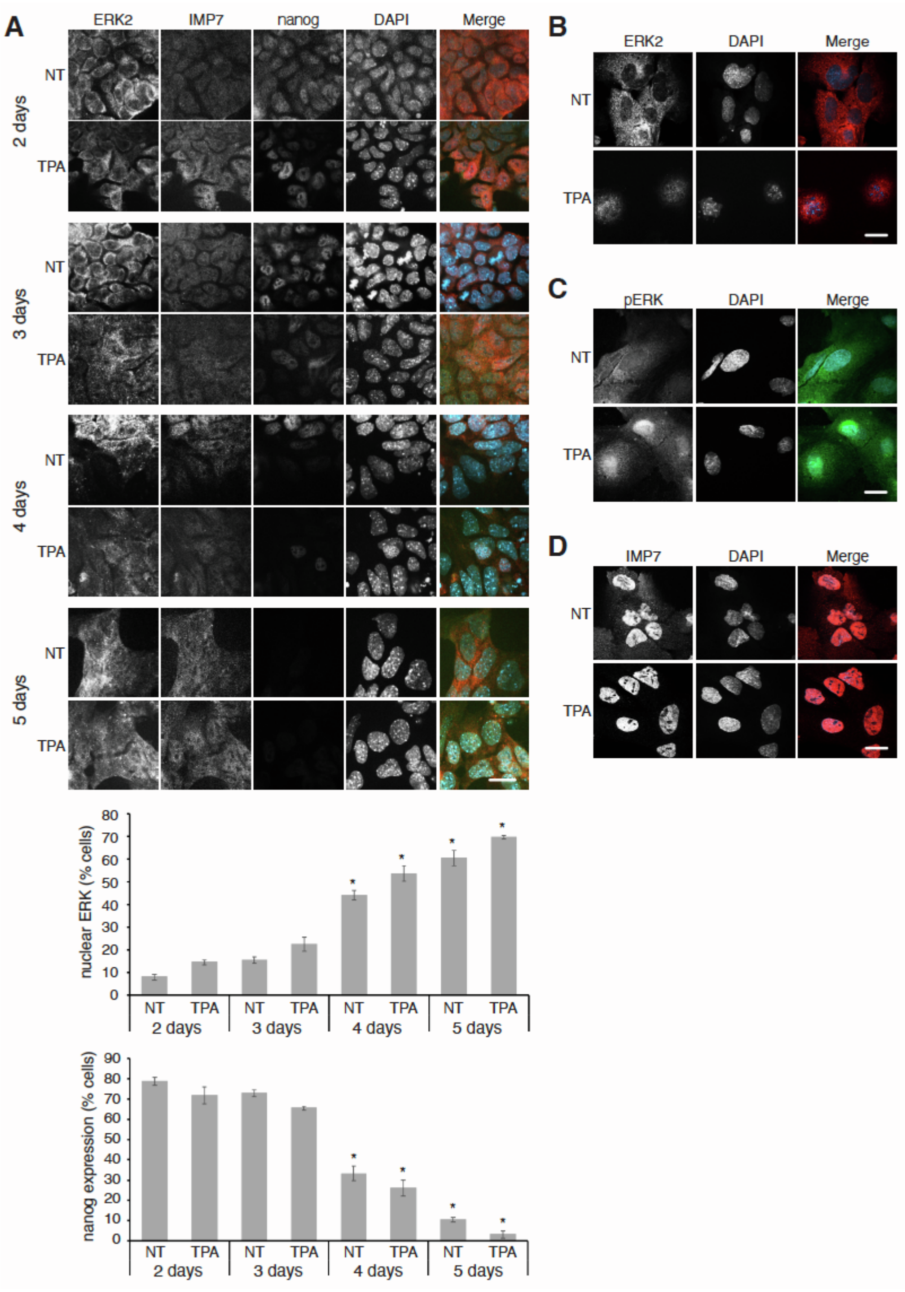

Figure S7: ERK localization during naïve to primed transition and in EpiSC, related to Fig. 5. (A) Expansion of figure 5. E14Tg2a cells were subjected to naïve to primed transition as in Fig. 5. In days 2, 3, 4 and 5 of the transition, some of the cells were stimulated with TPA (250 nM, 15 min), or left untreated (NT). The cells were then fixed and stained with the indicated Abs. Fluorescence was visualized by spinning disk confocal microscopy. The bar-graphs below represent percentage of cells that have nuclear ERK (left) and that express nanog (right). Means ± SE of three experiments. *$P < 0.01$ by paired T test indicates a significant change as compared with day 2. Scale bars - 20 μm in all **(B-D)** Nuclear translocation of ERK and IMP7 in EpiSCs. EpiSCs were produced from E14Tg2A for 6 passages. Fluorescence micrographs of ERK2 (B), pERK (C) and IMP7 (D) in these cells that were stimulated with TPA (250 nM 15 min) or left untreated (NT). Fluorescence was visualized by spinning disk confocal microscopy.

Supplemental Experimental Procedures

Reagents and antibodies. Tetradecanoyl phorbol acetate (TPA), epidermal growth factor (EGF), basic fibroblast growth factor (bFGF), anisomycin, CHIR99021, PD0325901, leptomycin B, insulin and 4,6-diamino-2-phenylindole (DAPI) were purchased from Sigma-Aldrich (Rehovot, Israel). FGF2 (100-18C) and Activin A (120-14) were from Peprotech (Rehovot Israel). Protein A/G PLUS-agarose beads were obtained from Santa Cruz Biotechnology, Inc. (CA, USA). Bovine serum albumin (BSA) was purchased from MP biomedical (OH, USA). ECL was from Biological Industries (Beit-Haemek, Israel). Lipofectamine2000 was from Thermo Fisher Scientific, (MA, USA). Recombinant human BMP4 from R&D Systems (Mn, USA). Anti-general (g) ERK1 (C-16) Cat #sc-93, anti-gERK2 (C-14) Cat #sc-154, anti-OCT3/4 Cat #sc-5279, anti-nanog Cat #sc-3769, anti-IMP7 Cat #sc-365231+ #sc-134913, anti-Elk1 Cat #sc-355, anti-pElk1 Cat #sc-8406, anti-RSK1 Cat #sc-231 , anti-JNK1/3 (C-17) Cat # sc-474, anti-IQGAP1 Cat #sc-376021 and normal IgG Cat #sc-68786 Abs were obtained from Santa Cruz Biotechnology (CA, USA). Anti-IMP7 Ab Cat #H00010527-M07 was obtained from Abnova (Taipei, Taiwan). Anti-pERK1/2 (p(TEY)ERK1/2) Cat #M8159, anti-gERK1/2 Cat #M5670, anti-gERK1 Cat #M7927 and anti-gMEK Cat #M5795 Abs were obtained from Sigma-Aldrich (Rehovot, Israel). Anti-Histone3 Cat #ab1791, anti-phospho cMyc Cat #ab51156 and anti-IMP7 Cat #ab15840 Abs were obtained from Abcam (Cambridge, UK). Anti-cMyc Cat #5605 and anti-pRSK (p90) Cat #9346S Abs were obtained from Cell Signaling (MA, USA) Anti-phospho SPS-ERK1/2 (1:500) Abs were produced in Biological Service Unit of the Weizmann Institute of Science (Rehovot, Israel). Secondary fluorescent Ab Cat #A10042, #A11008 were from Molecular Probes (Eugene, OR). Secondary Abs conjugated to horseradish peroxidase (HRP) Cat #111-035-003, #115-035-003 were obtained from Jackson ImmunoResearch laboratories (PA, USA)

The two peptides used were EPE - GQLNHILGILGEPEQED and Scramble—GNILSQELPHSGDLQIG (Plotnikov et al., 2015). Each of them was conjugated in its N-terminal to myristic acid and C-terminal amidated. The peptides were purchased from Gnenscript (NJ, USA). They were >85% pure and kept as 100 mM DMSO stock solution at −20 °C (Plotnikov *et al.*, 2015).

Buffers.

Buffer A - 50 mM β-glycerophosphate pH 7.3, 1.5 mM EGTA, 1 mM EDTA, 1mM dithiothreitol, and 0.1 mM sodium vanadate.

Buffer H – The same ingredients as Buffer A plus 0.1 mM sodium vanadate, 10 µg/ml aprotinin, 10 µg/ml leupeptin, 2 µg/ml pepstatin A, and 1 mM Benzamidin.

Radio-immunoprecipitation assay (RIPA) buffer - 137 mM NaCl, 20 mM Tris (pH 7.4), 10% glycerol, 1% Triton X-100, 0.5% deoxycholate, 0.1% SDS, 2 mM EDTA, 1 mM phenylmethylsulfonyl flouride (PMSF) and 20 µM leupeptin.

Co-IP Washing buffer – 20 mM HEPES pH 8.0, 2 mM $MgCl_2$, 2 mM EGTA, 100 mM NaCl.

Extraction buffer for the fractionation assay– 4 M NaCl, 1 M β-glycerophosphate, 0.1M Na_3VO_4, 0.2 M $MgCl_2$, 0.5 M EDTA, 1 M DTT, 25% glycerol.

Cell cultures.

The mESC lines E14Tg2a and V6.5 (Beard *et al*., 2006), were grown on plates coated with 0.2% gelatin in Glasgow Minimum Essential Medium (GMEM) supplemented with 15% fetal calf serum (FCS; Hyclone, ThermoFisher), 50 U/ml penicillin and streptomycin, 50 µg/ml glutamine, 1 mM Na Pyruvate, 0.1 mM β-mercaptoethanol, 1% MEM-Non-Essential Amino Acids (ThermoFisher) and 1000 U/ml Leukemia Inhibitory Factor (LIF; Merck, USA). For maintenance, V6.5 were cultured with irradiated mouse embryonic fibroblasts (MEFs; feeders) that were previously seeded on 0.1% gelatin. Primary (blastocyst-derived) mouse embryonic stem cells were produced with help from the Gross lab at the Weizmann Institute of Science (approved by the institutional Animal Care and Use Committee to the Gross lab). The cells were taken from pregnant mice, at 3.5 days post fertilization. The blastocysts were taken from the uterine horns, washed three times with the growing medium, and seeded on MEFs in a growing medium for a few days, until a mass of cells was formed, which are the embryonic stem cells. For maintenance, the cells were cultured with feeders and were grown in the same medium as E14Tg2a and V6.5 cells. ΔPE-OCT4-GFP transgenic reporter for pluripotency state of the mouse embryonic stem cells (Rais et al., 2013) were received from the Hanna's lab, Weizmann Institute of Science. The cells were grown in same condition as V6.5 cells. Their GFP levels were measured by FACS. 3T3-L1 cells were cultured in Dulbecco's modified Eagle's medium (DMEM) supplemented with 50 U/ml penicillin and streptomycin, 50 µg/ml glutamine and 10% fetal calf serum (FCS).

For naïve to primed and peptides experiments the cells were grown on plates coated growth factor reduced Matrigel (Corning, USA), in N2B27 medium contained DMEM-F12 and neurobasal at a 1:1 ratio supplemented with 0.5% N2 supplement (Gibco), 1% B27 supplement (Gibco), 50 U/ml penicillin and streptomycin, 50 µg/ml glutamine, 1 mM Na Pyruvate, 0.1mM β-

mercaptoethanol, 1% MEM Non-Essential Amino Acids All these reagents were from Gibco ThermoFisher. For naïve to primed transition, N2B27 media contained 12 ng/ml recombinant human FGF2 (Peprotech, Rehovot Israel) and 20 ng/ml recombinant human activin-A. In the peptides experiment, 3 μM CHIR99021 and recombinant mouse LIF 1000 U/ml were added to all conditions. PD0325901 1 μM, DMSO (1 μl/ml) or peptides 10 μM were added to each treatment. For serum free experiment, N2B27 media contained Recombinant human BMP4 (100 μg/ml) and recombinant mouse LIF 1000 U/ml for 2 passages. Mouse Embryonic Fibroblast (MEF) cells were grown on plates coated 0.2% gelatin in DMEM supplemented 50 U/ml penicillin and streptomycin, 50 μg/ml glutamine, 1 mM Na Pyruvate and 10% fetal calf serum (FCS). The "feeders" endow trophic factors that together with fetal calf serum and LIF support self-renewal. All cells were maintained at 37°C in a humidified atmosphere of 95% air and 5% CO_2.

EpiSCs were produced form E14Tg2A following the protocol published in reference (Brons et al., 2007). The cells were grown 2 passages with 2i+LIF media (N2B27 with 1 μM PD0325901, 3 μM CHIR99021 and LIF 1000 U/ml) and then transformed to N2B27 contained 1% FCS, FGF2 (12 ng/ml) and Activin A (20 ng/ml) for 4 passages. The cells were grown on plates coated growth factor reduced Matrigel for the whole procedure.

MEFs were prepared under sterile conditions. The pregnant female mouse (13.5 d.p.c.) was sacrificed. The uterine horns were dissected out and placed into a petri dish containing PBS. Each embryo was separated from its placenta and surrounding membranes and head, liver and gut were removed. The embryos were washed by transferred them to a petri dish containing clean PBS few times (to remove as much red blood cells as possible). Each embryo was homogenized with 1 ml medium by using 1cc syringe with an 18G 11/2" needle 7 times up and down. The suspension was plated on gelatinized plates, 1-1.5 embryos /10 cm plate in MEF medium. After four passages the cells were irradiated at 3000 rads. These cells were used as feeder for culturing primary mESCs.

Preparation of cell extracts and Western blotting. Cells were grown to subconfluence and serum-starved for 16 hr. After treatments, the cells were rinsed twice with ice-cold PBS and once with ice-cold Buffer A, scraped into Buffer H and disrupted by sonication (60 W, 2 × 7 sec) on ice or scraped into RIPA buffer. The extracts were centrifuged (15,000 rpm, 15 min at 4° C) and the supernatants were either subjected to co-immunoprecipitation or resuspended and boiled for 5 min in sample buffer. The samples were then subjected to 12% or 10% SDS-PAGE and Western blotting with the appropriate Abs, which were detected using alkaline phosphatase

or ECL according to the manufacturer's instructions. A representative Western blot of at least three independent experiments is shown for all cases.

TUNEL assay: in situ cell death was detected by using a TUNEL [terminal deoxynucleotidyl transferase (TdT)] kit (Roche Molecular Biochemicals). Cells were fixed in 4% paraformaldehyde in PBS (20 min, 23° C) and incubated with 2% BSA in PBS (15 min, 23°C), followed by permeabilization with Triton X-100 (0.1% in PBS, 5 min, 23° C). The fixed cells were washed again with PBS and incubated with terminal deoxynucleotidyltransferase-mediated nick end labeling (TUNEL) reaction mixture containing fluorescein-dUTP and terminal deoxynucleotidyl transferase for 30 min at 37° C. Preparations were analyzed by fluorescence microscopy. TUNEL staining of cells per total cell number was calculated. More than 100 cells were counted per treatment.

References

Beard, C., Hochedlinger, K., Plath, K., Wutz, A., and Jaenisch, R. (2006). Efficient method to generate single-copy transgenic mice by site-specific integration in embryonic stem cells. Genesis *44*, 23-28. 10.1002/gene.20180.

Brons, I.G., Smithers, L.E., Trotter, M.W., Rugg-Gunn, P., Sun, B., Chuva de Sousa Lopes, S.M., Howlett, S.K., Clarkson, A., Ahrlund-Richter, L., Pedersen, R.A., and Vallier, L. (2007). Derivation of pluripotent epiblast stem cells from mammalian embryos. Nature *448*, 191-195. 10.1038/nature05950.

Plotnikov, A., Flores, K., Maik-Rachline, G., Zehorai, E., Kapri-Pardes, E., Berti, D.A., Hanoch, T., Besser, M.J., and Seger, R. (2015). The nuclear translocation of ERK1/2 as an anticancer target. Nat Commun *6*, 6685. 10.1038/ncomms7685.

Rais, Y., Zviran, A., Geula, S., Gafni, O., Chomsky, E., Viukov, S., Mansour, A.A., Caspi, I., Krupalnik, V., Zerbib, M., et al. (2013). Deterministic direct reprogramming of somatic cells to pluripotency. Nature *502*, 65-70. 10.1038/nature12587.
