# Supplementary material for: Retention of ERK in the cytoplasm mediates the pluripotency of embryonic stem cells
Source: Stem Cell Reports. 2022 Dec 22;18(1):305–18. doi: 10.1016/j.stemcr.2022.11.017 (PMC9860118; doi:10.1016/j.stemcr.2022.11.017)
Supplement: Document S2. Article plus supplemental information [file mmc2.pdf]

Stem Cell Reports
Article
ISSCR
OPEN ACCESS

Retention of ERK in the cytoplasm mediates the pluripotency of embryonic stem cells

Avital Hacohen Lev-Ran[1] and Rony Seger[1,*]
[1]Department of Immunology and Regenerative Biology, Weizmann Institute of Science, Rehovot, Israel
*Correspondence: rony.seger@weizmann.ac.il
https://doi.org/10.1016/j.stemcr.2022.11.017

SUMMARY

The dynamic subcellular localization of ERK1/2 plays an important role in regulating cell fate. Differentiation of mouse embryonic stem cells (mESCs) involves inductive stimulation of ERK1/2, and therefore, inhibitors of the ERK cascade are used to maintain pluripotency. Interestingly, we found that in pluripotent mESCs, ERK1/2 do not translocate to the nucleus either before or after stimulation. This inhibition of nuclear translocation may be dependent on a lack of stimulated ERK1/2 interaction with importin7 rather than a lack of ERK1/2 phosphorylation activating translocation. At late stages of naive-to-primed transition, the action of the translocating machinery is restored, leading to elevation in ERK1/2-importin7 interaction and their nuclear translocation. Importantly, forcing ERK2 into the naive cells' nuclei accelerates their early differentiation, while prevention of the translocation restores stem cells' pluripotency. These results indicate that prevention of nuclear ERK1/2 translocation serves as a safety mechanism for keeping pluripotency of mESCs.

INTRODUCTION

The extracellular signal-regulated kinase 1/2 (ERK1/2) cascade mediates the signals of a variety of distinct stimuli to regulate many cellular processes, such as proliferation, differentiation, and more (Eblen, 2018; Lavoie et al., 2020; Maik-Rachline et al., 2019). To regulate these distinct functions, ERK1/2 are well regulated by phosphorylation, scaffolding, and subcellular localization (Kholodenko et al., 2010; Shaul and Seger, 2007). The subcellular localization of ERK1/2 is dynamic, as they are found in the cytoplasm of resting cells, but change their localization upon stimulation, reaching mainly the nucleus, but also the mitochondria, endosomes, cytoskeleton, and others (Watson et al., 2018; Wortzel and Seger, 2011). These changes in localization are important for proper cellular functioning upon stimulation and assist in the determination of cell fate. Importantly, the dysregulation of the cascade leads to a large number of distinct diseases, including cancer and developmental disorders (Lee et al., 2020).

The mechanism by which ERK1/2 are translocated to the nucleus was found to involve variable regulatory proteins (Flores et al., 2019). Thus, in resting cells, ERK1/2 are localized in the cytoplasm mainly owing to their interactions with a variety of anchoring proteins (e.g., MEK, β-arrestin, and SEF; Shaul and Seger, 2007). Upon stimulation, ERK1/2 are phosphorylated by MEK1/2, which causes both activation and detachment from the anchors (Chuderland et al., 2008). This detachment exposes the kinase insert domain of ERK1/2 and allows the phosphorylation of this region on two Ser residues within their nuclear translocation sequence (NTS; Plotnikov et al., 2019). The phosphorylation allows the binding of ERK1/2 to importin7 (IMP7) that escorts ERK1/2 to the nucleus via the nuclear pores. Importantly, a peptide (EPE) that interferes with ERK1/2-IMP7 interaction prevents nuclear ERK1/2 translocation and thereby inhibits proliferation of ERK-addicted cancer cells (Plotnikov et al., 2015).

The regulation of ERK1/2 activity has a significant influence on the pluripotent state of mouse embryonic stem cells (mESCs) (Weinberger et al., 2016). In these cells, the activation of ERK1/2 is implicated in the loss of pluripotency and differentiation of mESCs (Mossahebi-Mohammadi et al., 2020). Indeed, MEK inhibitors are routinely used for keeping mESCs in their self-renewal state (Dutta, 2013; Ying et al., 2008) combined with GSK3 inhibitor and LIF (2i medium). However, the effect of the subcellular localization of MEK1/2 and ERK1/2 on mESC fate is still missing (Morey et al., 2015; Weinberger et al., 2016). The main mechanism includes phosphorylation of transcription factors by ERK1/2, causing either their direct inhibition or the regulation of downstream factors (Kim et al., 2012, 2014; Meng et al., 2018). A recent study also showed that ERK1/2 reversibly regulate transcription in mESCs by directly affecting enhancer activity (Hamilton et al., 2019). Importantly, the effects of ERK1/2 on differentiation occur early in the process, as it was shown that the activity of the kinases is crucial for the progression from naive to primed state, which is the first step toward commitment in differentiation (Weinberger et al., 2016). Interestingly, although ERK1/2 signaling still contributes to self-renewal and genomic stability of naive cells even when their activity is reduced upon addition of MEK inhibitors (Chen et al., 2015), their full activity is essential for driving the primed pluripotency and further mESC differentiation (Hamilton and Brickman, 2014; Weinberger et al., 2016).

© 2022 The Author(s).
This is an open access article under the CC BY license (http://creativecommons.org/licenses/by/4.0/).

In this paper, we studied the role of ERK1/2 localization in pluripotency maintenance of the mESC. We found that ERK1/2 do not translocate to the nucleus of mESCs, even when the cells are stimulated by various extracellular agents. This is despite the high rate of proliferation of mESCs, which, in other cells, involves the activity of nuclear ERK (Maik-Rachline et al., 2019). This lack of translocation prevented the stimulated phosphorylation of nuclear transcription factor but not of cytosolic substrates. This lack of translocation is not because of a lack of ERK1/2 phosphorylation, but mainly due to the lack of interaction with or regulation by IMP7. Interestingly, the lack of translocation is reversed when the cells are advanced from naive to primed stages. We also found that forcing ERK2 into the nucleus accelerates the differentiation of mESCs, and inhibition of ERK1/2 translocation can be used to maintain mESC pluripotency. Overall, our results indicate that the prevention of nuclear ERK1/2 translocation is important for pluripotency maintenance of mESCs, which might be the way in which the mESC's pluripotency is maintained during early stages of embryogenesis.

RESULTS

ERK1/2 do not translocate into the nuclei of mESCs

Although the effect of ERK1/2 phosphorylation of transcription factors in mESCs is relatively well studied (Weinberger et al., 2016), much less is known about the role of ERK1/2 localization in this process. To study it, we first used common methods to follow the nuclear translocation (Chuderland et al., 2008; Rubinfeld et al., 1999). However, we were unable to use the standard serum starvation (0.1% fetal calf serum [FCS], 14–16 h), as this starvation imposed a significant stress that led to apoptosis of the mESCs (Figure S1). Shorter starvation times were not feasible because serum-induced expression of phosphatases is fully decayed only after ~14 h (Kraus and Seger, 2004). Searching for proper conditions, we found that 5% FCS + LIF (14–16 h) was the minimal serum concentration that did not cause changes in morphology, viability, or stimulation of the cells (Figures S1A and S1B). This condition was used in most of the experiments here. The starvation of 3T3-L1 cells was in the standard 0.1% serum to obtain maximal stimulation of this positive control. For stimulation, the cells were treated with stimuli that routinely induce nuclear ERK1/2 translocation (Chuderland et al., 2008; Plotnikov et al., 2015).

As ERK1/2's nuclear accumulation usually leads to proliferation (Michailovici et al., 2014), we expected to find nuclear accumulation of ERK1/2 in mESCs upon stimulation. Surprisingly, no nuclear ERK1/2 were detected in mESCs under any condition used. Thus, when E14Tg2a cells were maintained in 5% FCS + LIF, ERK1/2 were found solely in the cytosol and failed to translocate to the nucleus upon any of the stimulations used, which was different from the effects in the control 3T3-L1 cells (Figures 1A–1C). No nuclear translocation was observed either in the residual intact cells in 0.1% FCS stimulated with EGF (Figure S1C) or in starved cells (5% FCS) stimulated by adding 10% FCS (Figure S2A). This was also the case with cells grown in serum-free medium containing N2 and B27 supplements with LIF and BMP4 (Ying et al., 2003), even when we used FCS as a stimulator, or in cells grown constantly in 15% FCS + LIF (Figures S2B and S2C). Similar results were observed in different stem cell lines (V6.5 and primary [blastocyst-derived] stem cells, Figure S3), with a different time course of stimulation and staining with different ERK1/2 antibodies (Abs). These Abs also revealed that there is no difference in localization between ERK1 and ERK2 in these cells (Figures 1A and S4A). This occurred despite the active phosphorylated ERK1/2 (Figures 2A, S2D, S3B, and S3C). The lack of nuclear ERK1/2 localization both before and after stimulation can in principle be due to either a lack of translocation or a faster export out of the nucleus. To examine the export possibility, we used the exportin inhibitor leptomycin B (LMB) and found that it had no effect on the localization of ERK1/2 in E14Tg2a cells, irrespective of stimulation, when grown in either 0.1% FCS (Figure S1C) or 5% FCS (Figure S4B). This lack of nuclear translocation seems to be specific to ERK1/2, as we found that LMB retained MEK1/2, which use IMP7 for translocation (Chuderland et al., 2008) into the nucleus, when added with EGF (Figure S4D). This effect was somewhat different from that in other cells (Fukuda et al., 1997), in which LMB affects MEK localization even without stimulation. The reason that LMB does not cause nuclear accumulation without stimulation may indicate cytoplasmic anchorage of MEK1/2 in mESCs. We also found that JNK, which is similar in sequence to ERK1/2 and may use IMP7 for translocation (Maik-Rachline et al., 2018; Zehorai and Seger, 2019), did translocate upon EGF stimulation (Figure S5A). The function of MEK and JNK translocation upon EGF stimulation is not known, but indicates that the nuclear translocation machinery is still functional in the mESCs.

To further verify that ERK1/2 are active only in the cytoplasm of the mESC, we examined the phosphorylation of the nuclear transcription factors cMYC and ELK1 (Maik-Rachline et al., 2019) versus the cytosolic kinase RSK (Anjum and Blenis, 2008), which are all targets of ERK1/2 (Figure S5B). We found that the phosphorylation of the nuclear transcription factors was not changed upon stimulation. Since there was a small effect of the stimulation on the apparent expression of these two proteins (probably due to differential recognition by the Ab), the lack of

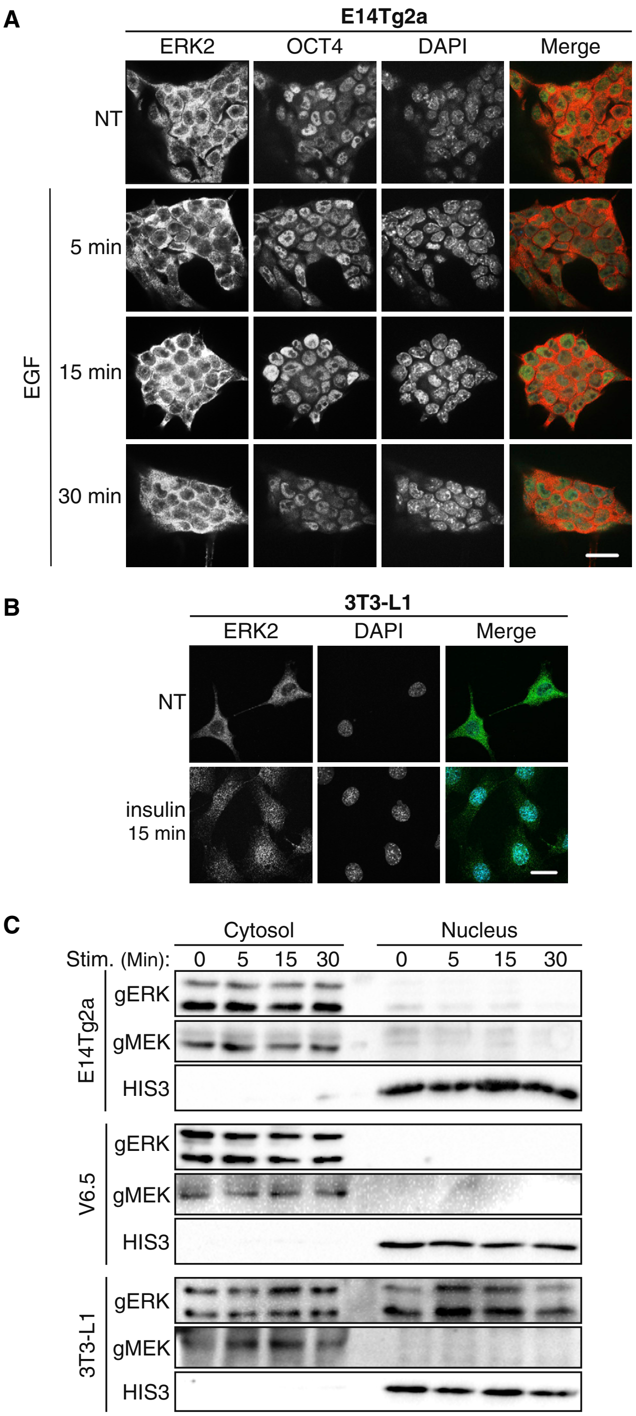

Figure 1. ERK1/2 do not translocate to the nucleus in mESCs
(A and B) Fluorescence microscopy. Images of E14Tg2a (A) and 3T3-L1 cells (B) stained with anti-ERK2 and anti-OCT4 Abs. The cells were grown in reduced serum concentration (5% for E14Tg2a cells or 0.1% serum in 3T3-L1 cells for 16 h) and then were either stimulated (EGF, 50 ng/mL, for the mESCs, and insulin, 100 nM, for the 3T3-L1 cells) for 5, 15, or 30 min or left untreated as control. The cells were then washed and stained with the indicated Abs. The nuclei were detected using 4′,6-diamino-2-phenylindole (DAPI). The fluorescence was visualized by spinning disk confocal microscopy. Scale bars, 20 μm.
(C) Subcellular fractionation of E14Tg2a, V6.5, and 3T3-L1 cells upon stimulation. The cells were grown in reduced serum concentration (16 h) and then were either stimulated with TPA (250 nM) for 5, 15, or 30 min or left untreated. After stimulation the cells were fractionated and then subjected to western blotting with the indicated Abs.
See also Figures S2–S4.

phosphorylation was shown by dividing the staining intensity of the phosphoprotein by that of the general protein (Figure S5B, bottom graphs). On the other hand, the stimulation did induce cytosolic pRSK. As expected, all three substrates were phosphorylated upon stimulation of the control 3T3-L1 cells. Based on these findings, and that nuclear ERK1/2 activity is required for mESC differentiation (Ying et al., 2008), it is likely that the lack of nuclear ERK1/2 contributes to the maintenance of the mESC's pluripotency.

ERK1/2 are phosphorylated on their TEY motif upon stimulation, while the pSPS motif is high in cycling cells and not elevated upon stimulation

We then undertook to study the molecular mechanisms involved in the lack of nuclear ERK1/2 translocation. One of the main mechanisms that allow ERK1/2 translocation is the TEY and SPS phosphorylation of ERK1/2 (Flores et al., 2019). Use of specific anti-pTEY and anti-pSPS ERK1/2 Abs revealed that TEY phosphorylation is increased upon stimulation (Figure 2A). On the other hand, the phosphorylation of the SPS was relatively high in resting cells and this was not significantly changed upon stimulation. This is different from the increase in SPS phosphorylation observed in the control 3T3-L1 (Figure 2A) and other cells (Plotnikov et al., 2015; Schevzov et al., 2015). The reason for the higher SPS phosphorylation at the basal stage compared with 3T3-L1 cells is not known but is clearly not sufficient to drive nuclear ERK1/2 localization (Figures 1 and S2–S4). Moreover, the fact that it is not significantly changed upon stimulation may be a reason for the lack of stimulated translocation. Importantly, staining with the anti-pTEY-ERK1/2 Ab showed that even the phosphorylated ERK1/2 are retained in the cytoplasm upon stimulation (Figure 2B).

To confirm that the lack of stimulated SPS phosphorylation plays a role in preventing the nuclear ERK1/2 translocation, we overexpressed ERK2 in mESCs (E14Tg2A) and found that the kinase is localized in the cytoplasm of resting cells (Figure 3). Overexpressed GFP-ERK2 can saturate cytoplasmic anchoring proteins and accumulate in the nucleus (Rubinfeld et al., 1999). Therefore, the

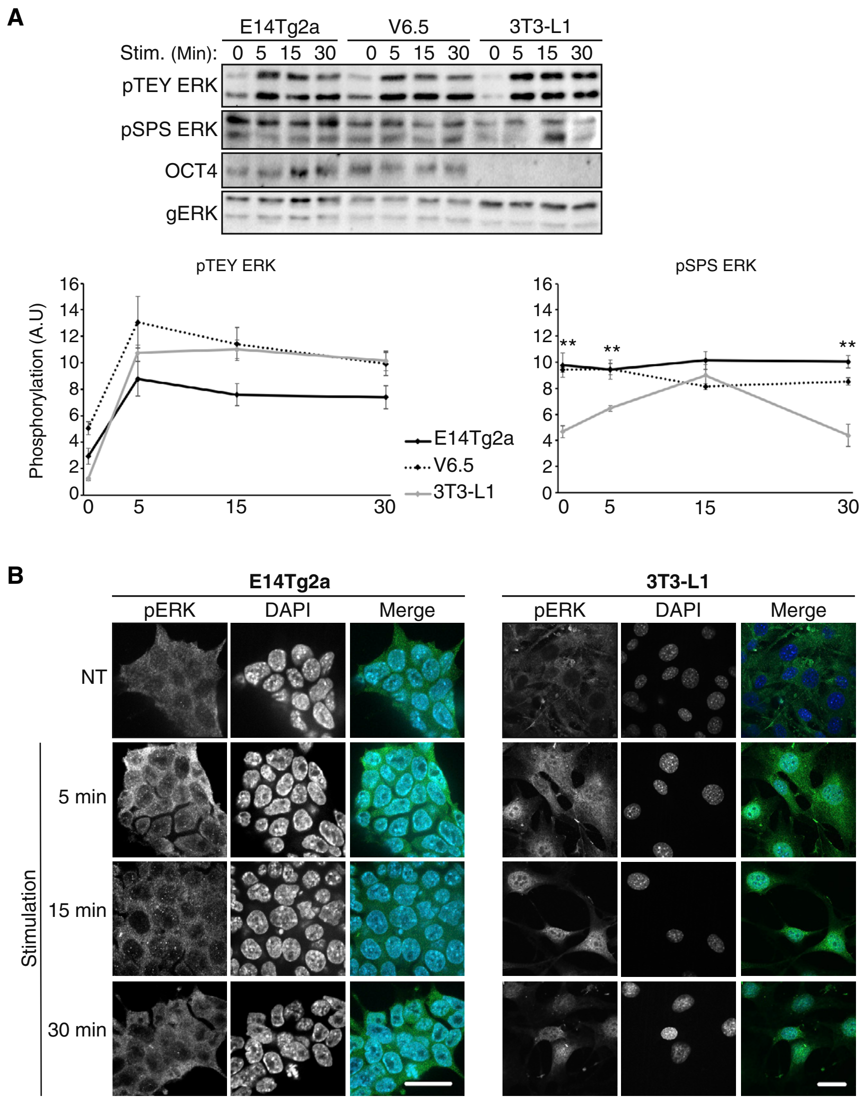

Figure 2. ERK1/2 phosphorylation on their TEY and SPS motifs in mESCs

(A) Phosphorylation of the TEY and SPS motifs of ERK1/2. E14Tg2a, V6.5, and 3T3-L1 cells were grown in reduced serum concentrations for 16 h (mESC, 5%; 3T3-L1, 0.1%) and then were either stimulated with TPA (250 nM) for 5, 15, or 30 min or left untreated (time 0). The cells were then harvested and subjected to western blotting using the indicated Abs. The results were then quantitated as appears in the lower graphs. The data are means ± SE of three experiments; $^{**}p < 0.01$, as calculated by Tukey's test, indicates significant changes between stem cell lines (E14Tg2a and V6.5) and the control cells (3T3-L1).

(B) Fluorescence microscopy of E14Tg2a or 3T3-L1 cells stained with anti-pERK1/2. Cells were grown in low serum concentration (E14Tg2a, 5%; 3T3-L1, 0.1%) for 16 h and then were either treated with EGF (50 ng/mL for the mESCs) or insulin (100 nM for the 3T3-L1 cells) for 5, 15, or 30 min or left untreated (NT). Next, the cells were fixed and stained with an anti-pERK-TEY Ab. The nuclei were detected using DAPI and the fluorescence was visualized by spinning disk confocal microscopy. Scale bars, 20 μm.

See also Figure S5.

cytoplasmic localization observed in both the mESC and the 3T3-L1 might be due to excess cytoplasmic anchoring. We also used an ERK2 mutant in which the Ser residues in the SPS motif were replaced with the phosphomimic residue Glu (EPE mutant). This mutant was previously shown to translocate to the nucleus and stay in that organelle even without any stimulation (Chuderland et al., 2008). Interestingly, this mutant also was localized exclusively in the mESCs' cytoplasm, which was different from the control 3T3-L1 cells. Importantly, overexpressed ERK2 with a canonical NLS conjugated to its C terminus (ERK2-NLS), which is found in the nucleus of cells independent of stimulation (Casar et al., 2012), was also found in the nucleus of both E14Tg2A and 3T3-L1 cells. This indicates that ERK2 preserves its nuclear shuttling ability in mESCs, and the lack of shuttling is due to other mechanisms.

Role of cytoplasmic anchoring proteins in preventing nuclear ERK1/2 translocation

Phosphorylation of the SPS motif occurs after the release of ERK1/2 from cytoplasmic anchoring proteins, provided that the interaction is mediated by the common docking motif of ERK1/2 (CD) and a D domain of the anchor (Plotnikov et al., 2019). However, other protein interaction sites in ERK1/2 exist as well (Eblen, 2018), which are not reversible upon stimulation, and therefore the interaction with these proteins does not allow SPS phosphorylation. It was possible that the lack of translocation in mESCs was due to irreversible binding to an abundant cytoplasmic anchor(s). To test it, we knocked down putative anchoring proteins (Figure S6), including GAB1, DAG1, and FHL2, whose expression is changed in stem cells (Kojima et al., 2014). We also knocked down

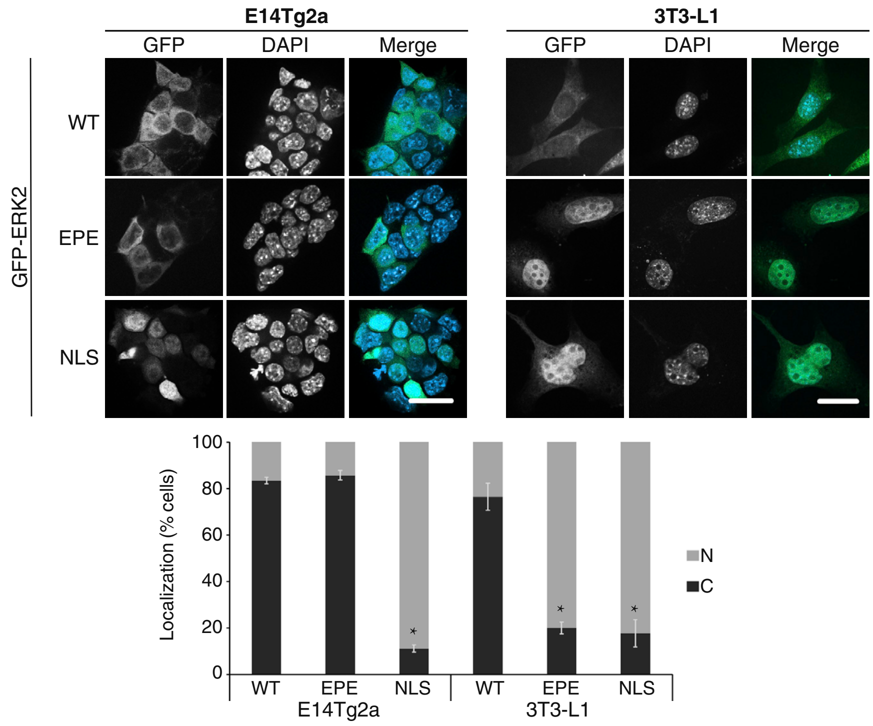

Figure 3. Lack of WT and ERK2-EPE accumulation in the nuclei of E14Tg2a cells

E14Tg2a and 3T3-L1 cells were transfected with GFP-ERK2, GFP-ERK2-EPE, and GFP-ERK2-NLS and stained with DAPI to detect the cells' nuclei, and the fluorescence was visualized by spinning disk confocal microscopy. Percentage of cells with mostly nuclear (N, gray) or mostly cytoplasmic (C, black) is shown in the bar graph below. Fifty cells were counted in each experiment, and data are means ± SE of three experiments. *$p < 0.05$ as calculated by t test. Scale bars, 20 μm. See also Figure S6.

PEA-15, MORG1, and IQGAP1, which are known anchoring proteins in other cells (Wortzel and Seger, 2011). Despite the pronounced reduction (more than 70%), these knockdowns had no effect on the subcellular localization of the ERK1/2. Since we examined the most probable anchors, and with the fact that GFP-ERK2-NLS does translocate to the nucleus (Figure 3), we concluded that anchoring interactions are probably not the main component involved in the lack of nuclear ERK translocation in mESCs. However, since more than 30 ERK1/2 anchoring proteins have been reported by now (Wortzel and Seger, 2011), we cannot rule out the possibility that other, less expected, proteins or their combination plays some role in the process.

Role of IMP7 in preventing nuclear ERK1/2 translocation

Another possibility for the inability of ERK1/2 to translocate to the nucleus might be a lack of interaction with IMP7 (Chuderland et al., 2008). The ability of NLS-ERK2 to accumulate in the nucleus implies that the effect is specific to IMP7 and not other importins. Indeed, it was previously reported that IMP7 plays a role in the differentiation of mESCs (Sangel et al., 2014), and we examined here whether this may be executed via its interaction with ERK1/2. Mechanisms by which IMP7 may be dysregulated are either reduced IMP7 expression or reduced interaction with ERK1/2. As for the first, we found that the expression of IMP7 in the mESC is roughly similar to that 3T3-Ll cells (Figure 4A), which is supported by a previous publication showing that IMP7 is expressed in mESCs similar to or slightly more than in other cells (Sangel et al., 2014). However, unlike 3T3-L1 control cells, no interaction between ERK1/2 and IMP7 was detected in mESCs (Figures 4A and 4B), suggesting that this is indeed the influencing mechanism.

Unfortunately, the regulation of the interaction is not fully understood. However, it may involve changes in localization, as IMP7 was previously shown to accumulate in the nucleus upon stimulation (Zehorai and Seger, 2019). We next examined the subcellular distribution of IMP7 in mESCs compared with 3T3-L1 cells. A fractionation experiment revealed no significant changes in the amount of IMP7 in the cytoplasm or nucleus of the mECS, while it was enriched in the nuclei of the control 3T3-L1 cells upon stimulation (Figure 4C). Similar results were obtained using immunofluorescent staining (Figure 4D) showing that IMP7 is localized all over the cell, both before and after stimulation with the phorbol ester TPA, which causes receptor-independent activation of ERK1/2. This was unlike control 3T3-L1 and HeLa cells (Zehorai and Seger, 2019), in which IMP7 translocated to the nucleus upon stimulation. We also found that IMP7 localization was not changed upon EGF stimulation (Figure S4C), but some nuclear accumulation was seen upon LMB treatment. Therefore, it is likely that IMP7 can translocate to the

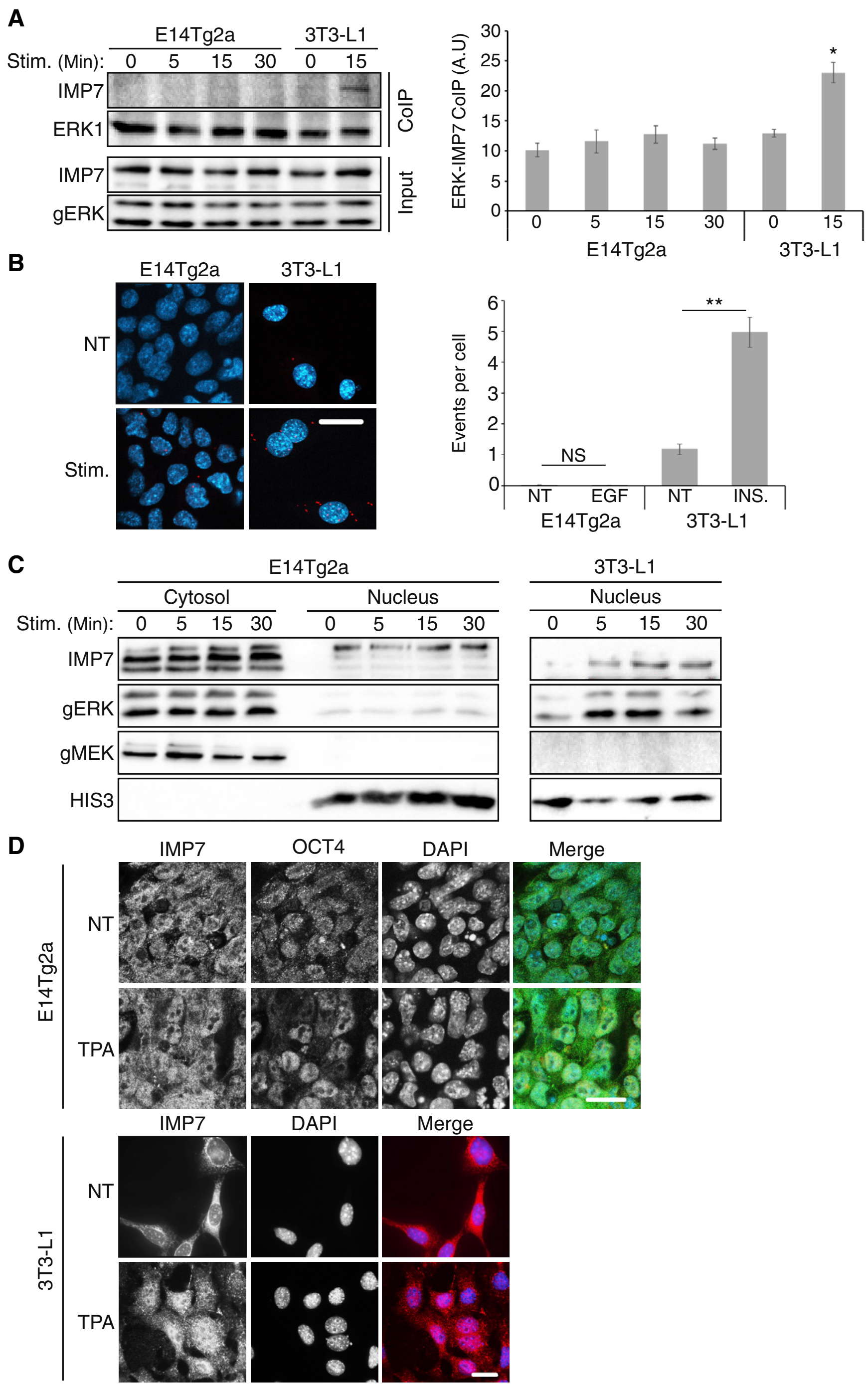

Figure 4. The subcellular localization and ERK1/2 interaction of IMP7

(A) Coimmunoprecipitation (coIP) of IMP7 with ERK1 in E14Tg2a and 3T3-L1 cells. The cells were grown in reduced serum concentrations (E14Tg2, 5%; 3T3-L1, 0.1%) for 16 h and either stimulated with TPA (250 nM) for 5, 15, or 30 min or left untreated (0). The cells were then harvested and subjected to coIP with anti-ERK1/2 Ab followed by washes with coIP buffer. Finally, the beads used for coIP were boiled in sample buffer and subjected to western blotting with the indicated Abs. The loading extracts (bottom) were blotted as well. The bar graph on the right represents means ± SE of three experiments. $^*p < 0.05$, as calculated by Tukey's test.

(B) PLA using anti-ERK2 and IMP7 Abs of E14Tg2a or 3T3-L1 cells. The cells were grown as described above and then were stimulated (EGF, 50 ng/mL, for the mESCs, and insulin, 100 nM, for the 3T3-L1 cells) for 5, 15, or 30 min or left untreated. The cells were then fixed and subjected to PLA and stained with DAPI to detect nuclei. The fluorescence was visualized by spinning disk confocal microscopy. Scale bar, 20 μm. The bar graph on the right represents means ± SE of three experiments. $^{**}p < 0.01$; NS, not significant; as calculated by Tukey's test.

(C) IMP7 localization using subcellular fractionation of E14Tg2a and 3T3-L1 cells. The cells were grown as described above and then stimulated (EGF, 50 ng/mL, for the mESCs, and insulin, 100 nM, for the 3T3-L1 cells) for 5, 15, or 30 min or left untreated (0). The cells were then harvested and subjected to a subcellular fractionation followed by a western blot analysis using the indicated Abs.

(D) Fluorescence microscopy of IMP7 in E14Tg2a cells or 3T3-L1 cells. The cells were grown as above and stimulated with TPA (250 nM, 15 min) or left untreated (NT), fixed, and stained with anti-IMP7 and anti-OCT4 Abs. The nuclei were detected using DAPI. The fluorescence was visualized by spinning disk confocal microscopy. Scale bars, 20 μm.

nucleus, but unlike in TPA- or EGF-stimulated HeLa and 3T3-L1 cells, it is exported back to the cytoplasm. Thus, it is likely that the regulation of IMP7 in mESCs is different from that in other cells, but more studies are required to fully understand it.

Changes in ERK1/2 translocation during naive-to-primed transition and in mESC-differentiated EPiSCs

mESCs can be found in either naive or primed states (Weinberger et al., 2016), where the naive state is the more pluripotent and the primed state is a first step toward

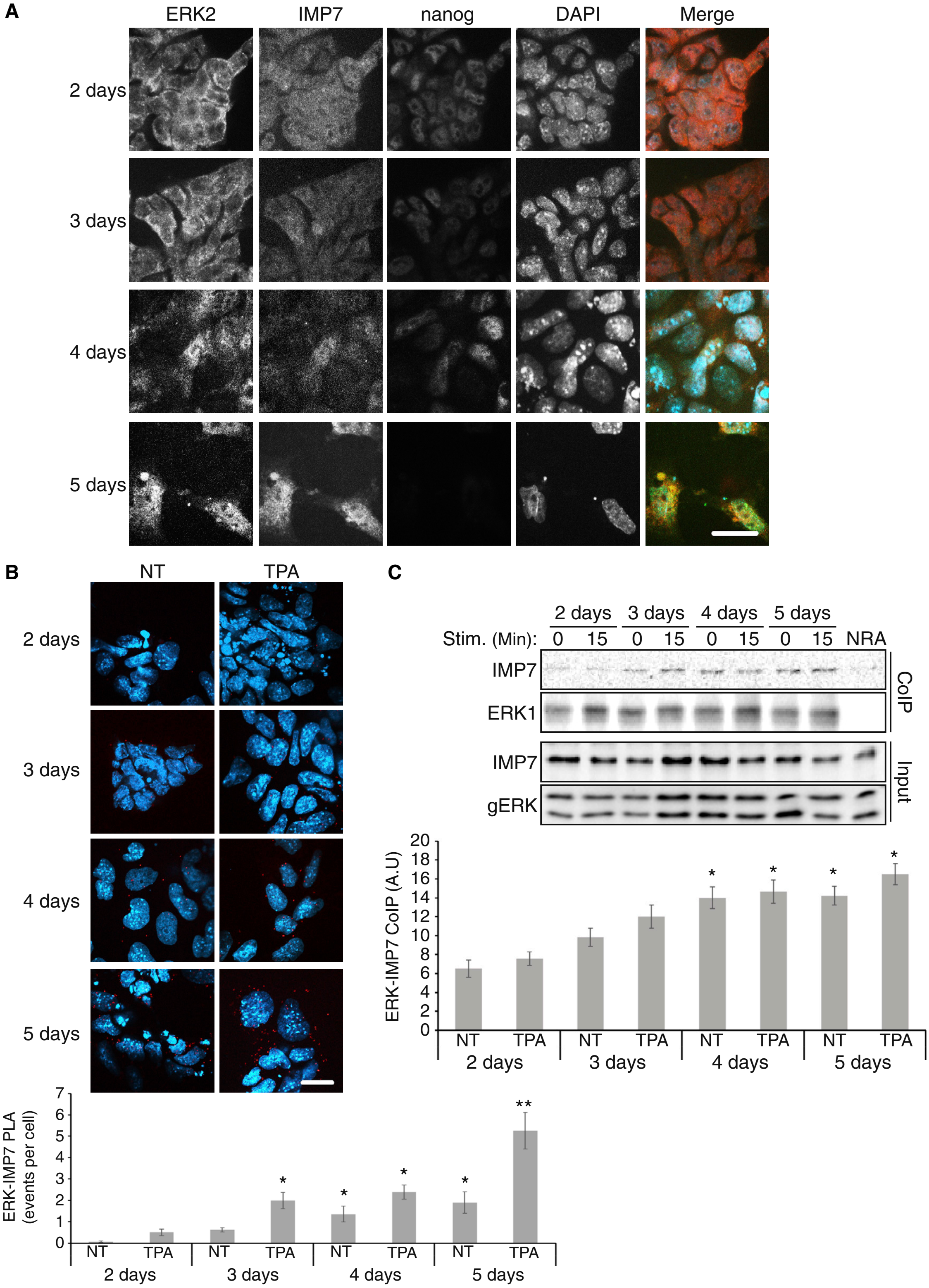

(legend on next page)

differentiation, during which the mESCs undergo epigenetic changes (Hackett and Surani, 2014). It was shown that inhibition of ERK1/2 is required for pluripotency, while their activation is important for the primed and more differentiated states. We undertook to study whether the subcellular localization of ERK1/2 is affected during the transition. Therefore, we followed ERK1/2 localization in the mESCs during transition, with or without stimulation. We also followed IMP7's localization and ERK-IMP7 interaction. Naive-to-primed transition was achieved by adding FGF2 and activin to N2B27 medium for up to 5 days. Immunofluorescent staining revealed that in days 2 and 3 of transition, ERK1/2 and IMP7 distribution is not significantly different from that of the cytoplasmic ERK1/2 or the all over distribution of IMP7 in naive cells (Figure 5). Interestingly, at day 4, there was a small shift into nuclear ERK1/2 localization, and on day 5, the changes were more significant, as ERK1/2 were localized either all over the cell or in the nucleus, and IMP7 was mostly nuclear. Stimulation of the cells with TPA had no significant effect on the localization (Figure S7A). The changes in subcellular localization during naive-to-primed transition were accompanied by elevated ERK1/2-IMP7 interaction (Figures 5B and 5C), indicating that the ERK1/2 translocation system is recovered during the process. Again, TPA stimulation did not significantly change the ERK-IMP7 interaction, suggesting that most of the ERK1/2 molecules capable of translocation were already nuclear and could not be further changed by TPA. Interestingly, at day 5, the proximity ligation assay (PLA) showed an increase in interaction, despite the lack of change using coimmunoprecipitation (coIP). The reason for this change is not clear, but since there is no effect on the stimulated nuclear translocation, it does not justify further study.

To further study the localization, we also used E14Tg2A-produced epiblast stem cells (EPiSCs) (Brons et al., 2007), which are more differentiated than the primed cells. Despite the large amount of FGF2 in the growth medium, ERK1/2 and phosphorylated ERK1/2 (pERK1/2) were localized in the cytoplasm before stimulation and shifted to the nucleus upon stimulation (Figures S7B and S7C). IMP7 in these cells was mostly nuclear, but a small amount was found in the cytoplasm of unstimulated cells, which shifted to the nucleus upon stimulation (Figure S7D). Overall, our results indicate that the mechanisms that prevent nuclear ERK1/2 translocation, namely lack of an interaction with IMP7 and lack of IMP7 translocation, are changed during naive-to-primed transition. This might be one of the first steps in mESC differentiation, as seen in EPiSCs and all other cell lines tested by now (Maik-Rachline et al., 2019).

Forcing ERK2 into the nucleus accelerates pluripotency marker disappearance, while prevention of translocation maintains mECS pluripotency

To examine the importance of the nuclear ERK1/2 on the differentiation of mESCs, we forced ERK2 into the nucleus. For this purpose, we used ERK2-NLS, which is found constantly in the nucleus. The transfected mESCs in N2B27 medium were treated for 2 days with FGF2 and activin to allow the initial stage of naive-to-primed transition. Then, the cells were stained for nanog to follow differentiation. As in the naive-to-primed transition (Figure S7A), 20% of GFP-ERK2 (wild type; WT) transfected cells lost their nanog. However, 44% of ERK2-NLS-expressing cells lost nanog expression, indicating a faster exit from pluripotency (Figure 6A), equivalent to day 4 of the naive-to-primed transition. These findings were similar to those for OCT4 (Figure 6B), which, due to its slower disappearance compared with nanog during differentiation (Papatsenko et al., 2015), was tested after 5 days.

Then, we examined whether inhibition of nuclear ERK1/2 translocation keeps the cells in a pluripotent stage and can be used instead of MEKi for maintaining

Figure 5. ERK1/2 and IMP7 localization and interaction in the naive-to-primed transition

(A) Fluorescence microscopy of ERK2, IMP7, and nanog in E14Tg2a cells during naive-to-primed transition. Cells were induced to undergo naive-to-primed transition for 5 days by transferring them to N2B27 medium with FGF2 (12 ng/mL) and activin A (20 ng/mL). On days 2, 3, 4, and 5 of the transition, the cells were fixed and stained with the indicated Abs and with DAPI. Scale bar, 20 μm.

(B) PLA of ERK1/2 and IMP7 interaction during transition. E14Tg2a cells were subjected to naive-to-primed transition as above. On each day the cells were either stimulated with TPA (250 nM, 15 min) or left untreated and then the cells were fixed and subjected to PLA using anti-ERK2 and anti-IMP7 Abs. The fluorescence was visualized by spinning disk confocal microscopy. Scale bar, 20 μm. The data presented in the bar graph below represent means ± SE of three experiments. *$p < 0.05$ and **$p < 0.01$ as calculated by Tukey's test indicate a significant change compared with 2 days.

(C) coIP of IMP7 with Ab to ERK1 in E14Tg2a during naive-to-primed transition. The cells were treated as above. Each day, the cells were harvested and subjected to coIP using anti-ERK1 Ab. Then the beads were boiled in sample buffer that was further subjected to a western blot analysis with the indicated Abs. The extracts were blotted as well, as seen in the bottom. The results in the bar graph below represent means ± SE of three experiments. *$p < 0.05$ by Tukey's test indicates a significant change compared with 2 days.

See also Figure S7.

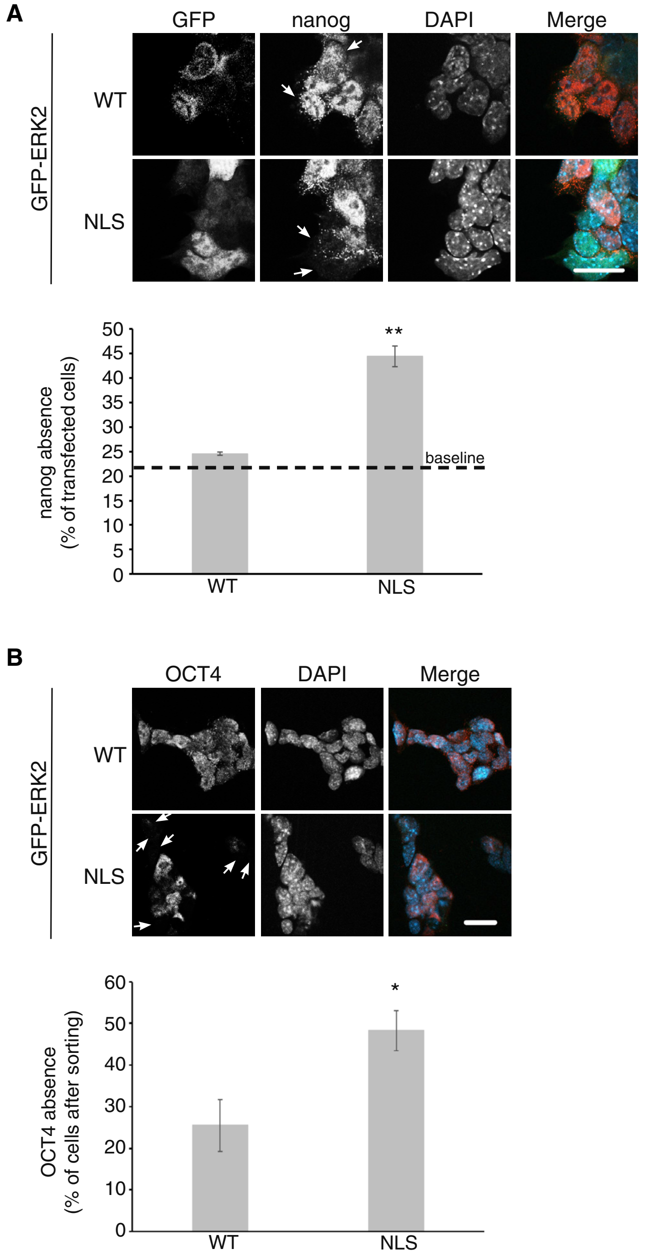

Figure 6. Forcing ERK2 into the nucleus accelerates exit from pluripotency of mESCs

E14Tg2a cells were transfected with constructs expressing GFP-ERK2 or GFP-ERK2-NLS.

(A) The cells were grown in N2B27 medium with FGF2 (12 ng/mL) and activin A (20 ng/mL) for 2 days and then fixed and stained with anti-nanog and DAPI, and the fluorescence was visualized by spinning disk confocal microscopy. GFP- and nanog-expressing cells were counted. The arrows in the nanog staining indicate cells lacking nanog. Scale bar, 20 μm. The bar graph shows percentage of transfected cells without nanog. The dashed line presents the part of cells that loss their nanog after the 2 days of treatment (as in Figure S7). Fifty cells were counted in each experiment.

(B) Because OCT4 disappears after a longer time, in such a way that the GFP cannot be observed anymore, we used FACS to select transfected cells 62 h after transfection. These cells were further raised in N2B27 for an additional 2 days (total 110 h), fixed, and stained with anti-OCT4 and DAPI. Scale bar, 20 μm. The arrows in the bottom left image indicate cells lacking OCT4. The bar graph shows percentage of OCT4-lacking cells. One hundred fifty cells were counted in each experiment. The graphs represent means ± SE of three experiments. $^{**}p < 0.01$ and $^{*}p < 0.05$, as calculated by t test.

pluripotency (Morey et al., 2015). MEKi blocks almost exclusively ERK1/2 signaling to allow pluripotency (Ma et al., 2016). Since we found that the lack of nuclear ERK1/2 may lead to pluripotency, we examined the effect of preventing nuclear translocation. For this purpose, we used the EPE peptide, which attenuates ERK1/2 translocation in all cells examined and reduces proliferation of various cancer cells (Plotnikov et al., 2015). This peptide and its scrambled control (SCR) were added to the mESCs in 2i medium instead of MEKi for 10 passages. Then we analyzed the cells' stemness characteristics, including changes in colony morphology and number, differentiation, and OCT4 expression of the cells. We found that the cells grown with the EPE peptide are similar to the cells grown in the regular 2i medium, while in the cells grown with SCR peptide or DMSO, many colonies started to differentiate (Figures 7A and 7B). In addition, the numbers of the colonies formed in the 2i or EPE-peptide-treated cultures were similar, while in the controls (SCR peptide and DMSO) the number was reduced (Figure 7C). This indicates that, similar to 2i, the EPE peptide prevented the differentiation. To further confirm this point, we used mESCs containing a stringent ΔPE-OCT4-GFP pluripotency reporter (Rais et al., 2013). These cells were grown with 2i, EPE peptide, or SCR peptide control and then analyzed by fluorescence-activated cell sorting (FACS) to detect GFP-containing pluripotent cells. We found that in the SCR group there were more cells that did not express GFP (lost their pluripotency) than in the EPE and 2i groups (Figure 7D). This further confirms that nuclear ERK1/2 translocation is important for maintaining mESC pluripotency, and prevention of this translocation can mimic the effect of MEK inhibitor in maintaining these cells.

DISCUSSION

In mESCs, the ERK1/2 cascade is known to affect cell fate, inducing exit from pluripotency to differentiation (Kim et al., 2013). Thus, a lack of ERK1/2 activity is critical for the maintenance of pluripotency, and indeed, treating the cells with a MEK inhibitor is a very common method

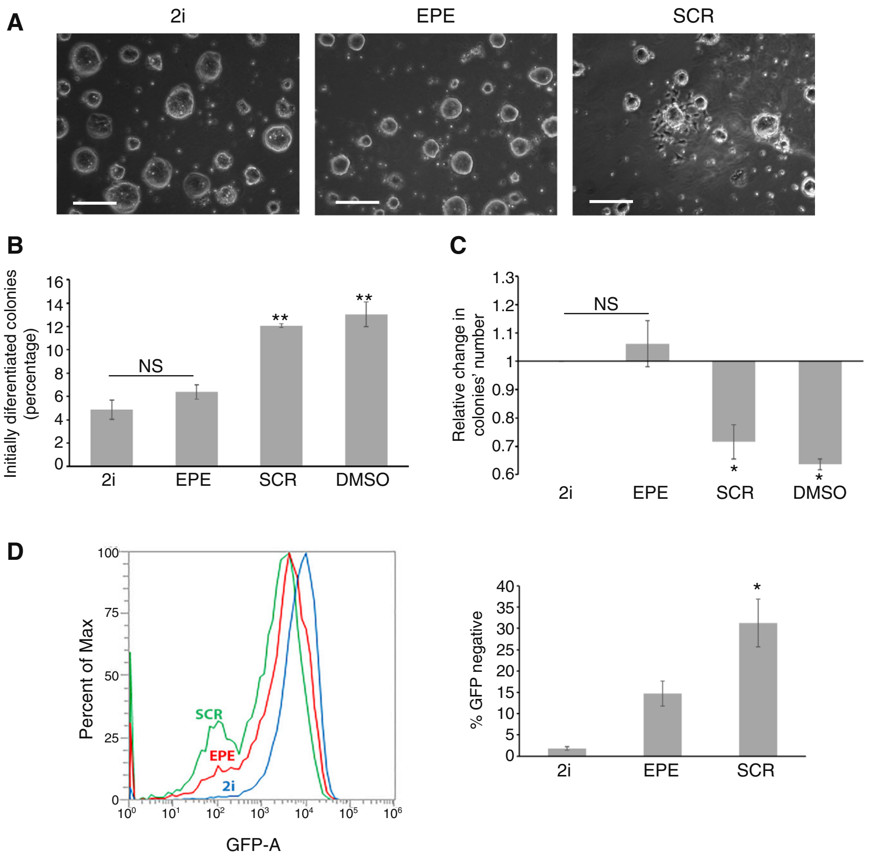

Figure 7. Using ERK1/2 translocation inhibitor instead of MEKi for mESC pluripotency

(A–C) Pluripotency of mESCs is maintained by 2i and EPE peptide. E14Tg2a cells were grown in N2B27 with GSK3i (CHIR99021, 3 μM), LIF (1,000 U/mL), and MEKi (PD0325901, 1 μM) (left), EPE peptide (10 μM) (middle), or scramble peptide (10 μM) (right). (A) The colony formation and numbers were detected by regular light microscopy. Scale bars, 500 μm. (B) Quantification of the results in (A) as the percentage of colonies with initial differentiation, which was defined by losing the round shape of the colony and the appearance of single cells around it. The bar graph represents the average and SE of 150 colonies in three experiments. $^{**}p < 0.01$ by Tukey's test. (C) Another quantification of (A) done by the relative changes in colony number compared with the colonies formed in 2i. The quantifications were done on 60 fields for each condition, including DMSO (1 μL/mL). Data are the mean ± SE of three distinct experiments. $^{**}p < 0.01$, $^{*}p < 0.05$; NS, not significant by Tukey's test.

(D) Loss of pluripotency determined by ΔPE-OCT-GFP. ΔPE-OCT-GFP-containing cells were grown in N2B27 with GSK3i (CHIR99021, 3 μM), LIF (1,000 U/mL), and MEKi (PD0325901, 1 μM), EPE peptide (10 μM), or scramble peptide (10 μM). After 2 days the cells were subjected to FACS analysis to determine GFP expression. The bar graph on the left represents mean ± SE of the percentage of negative cells (lost pluripotency) of three experiments; $^{*}p < 0.05$ as calculated by Tukey's test.

in mESC culturing (Ying et al., 2008). The molecular mechanisms that are involved in ERK1/2-regulated self-renewal of mESCs include phosphorylation and modulation of the activity of transcription factors and enhancers that are involved in this process (Hamilton et al., 2019; Meng et al., 2018; Ochiai et al., 2020). However, the determination of ERK1/2's specificity and the interaction with other signaling pathways remain elusive. Considering the pivotal role of ERK1/2 in mESCs, we undertook to study the role of the subcellular localization of ERK1/2 in determining the mESC fate. To our surprise, we found that in all mESCs examined (E14Tg2a, V6.5, and primary), ERK1/2 did not translocate to the nucleus upon any stimulation examined.

Very little is known to date about ERK1/2 localization in mESCs. Indeed, it was previously shown that endogenous pERK is observed solely in the cytoplasm of mESCs, but some ERK1/2 activity can be detected also in the nucleus (Deathridge et al., 2019). However, this point was not confirmed, since: (1) no ERK1/2 phosphorylation was shown in the nucleus; (2) overexpression might lead to erroneous localization, which was not examined here; (3) the reporter used might be phosphorylated by other kinases downstream of ERK1/2; and (4) the biosensor itself could transfer ERK1/2 to the nucleus by its conjugated NLS. Another study that claimed that active ERK1/2 may be localized in the nucleus of mESCs used fractionation as the main readout for nuclear accumulation (Dhaliwal et al., 2018). However, this was problematic as: (1) it failed to detect any cytoplasmic pERK1/2, which is very unlikely according to the results obtained by us and others; (2) in contrast to common knowledge (Shaul and Seger, 2007), the authors claimed that ERK1/2 bind with exportin1, which contradicts the lack of cytoplasmic ERK1/2 in that study; and (3) the maintenance medium in that study contained MEKi, which affects ERK1/2 activity and might be the reason for the difference in results. These studies and our findings above led us to study the mechanisms that enable the lack of translocation in regulating pluripotency.

We found that the lack of translocation in the mESC does not include a non-reversible anchorage in the cytoplasm, but does involve interaction of the SPS-phosphorylated ERK1/2 with the nuclear importer IMP7.

Interestingly, despite the relatively high level of SPS phosphorylation, we detected no ERK-IMP7 interaction, either before or after stimulation (Figure 4). This result is supported by the fact that the phosphomimetic mutant (EPE) of ERK1/2 was unable to translocate to the nucleus as well. We believe that this lack of interaction is mediated by dysregulation of IMP7 and is probably the main reason for the lack of nuclear ERK1/2 translocation. The fact that IMP7 is dysregulated in mESCs is also supported by the fact that this protein does not change its localization upon stimulation, as is apparent in 3T3-L1 cells (Figure 4). Not enough information on IMP7 regulation in either resting or stimulated cells exists in this stage, and therefore, it is difficult to assess the exact changes in the mESC. However, a clue to such a change can be derived by the very specific anti-Imp7 Abs that detect three distinct bands in E14Tg2a, unlike the one band in the 3T3-L1. The nature of these bands is not known, but might be due to distinct (unknown) alternatively spliced isoforms or posttranslation modification. It is possible that the modified form(s) of IMP7 that is expressed in mESCs is unable to interact with pERK1/2 and drive their nuclear translocation. However, expression of alternative splicing or phosphorylated IMP7 needs further studies.

In mature mouse cells, ERK1/2 do translocate to the nucleus upon stimulation, which means that during differentiation, the mechanism that prevents ERK1/2 from translocating either disappears or changes to allow translocation. While naive mESCs have no ERK1/2 activity, the transition to the primed state is dependent on ERK1/2 activation (Hackett and Surani, 2014). Interestingly, a study of mouse blastocyst showed that around day 3.25–3.75, pERK1/2 is localized exclusively in the cytoplasm, while on day 4.5, when the cells start to differentiate, pERK is seen in the nucleus as well (Azami et al., 2019), supporting our results that the naive-to-primed transition regains nuclear ERK1/2 translocation. Importantly, we also show that forcing ERK into the nucleus accelerates the differentiation, while prevention of the nuclear ERK1/2 translocation can keep mESCs in their pluripotent state. These results also show that nuclear ERK1/2 translocation is required for mESC differentiation. These are unlike other studies in which cytoplasmic ERK1/2 correlated with differentiation (Formstecher et al., 2001; Michailovici et al., 2014). Thus, our study and others (e.g., Herrero et al., 2015) indicate that the effect is cell-type specific. However, the exact mechanism that mediates the effects in mESCs and other cells requires further clarification. Moreover, translocation inhibitors such as the EPE peptide (Plotnikov et al., 2015) can be used instead of MEKi to keep the cells in the pluripotent state. Although the EPE peptide was less effective than MEKi, it kept part of its activity. The lower effect of the peptide could be because the peptide is not as good an inhibitor as the small molecule MEKi. Nonetheless, our results clearly indicate that the nuclear translocation of ERK1/2 does play a role in the exit from the pluripotent state of mESCs, and this point should be further studied.

In summary, ERK1/2 do not translocate to the nucleus upon stimulation in mESCs. This is probably due to dysregulation of IMP7 and the reduced IMP7-ERK1/2 interaction. Our observations led us to hypothesize that prevention of the ERK1/2 translocation serves as a safety mechanism for proper self-renewal of native embryonic stem cells. This is reversed in the first step toward differentiation (naive-to-primed transition), which eventually allows translocation of ERK1/2. Keeping the cells in the pluripotent state by interrupting with ERK-IMP7 interaction strengthens our hypothesis and indicates that nuclear exclusion of ERK1/2 may serve as a safety mechanism to prevent differentiation of mESCs in native conditions during embryonic development.

EXPERIMENTAL PROCEDURES

Resource availability

Corresponding author

The corresponding author for this work is Rony Seger (rony.seger@weizmann.ac.il).

Materials availability

All materials we generated will be made available upon request.

Data and code availability

The original uncropped blots and FACS data are deposited in Mendeley Data: https://doi.org/10.17632/x4pdkhhxkb.1. This paper reports no original code.

DNA constructs, knockdown by siRNA, and transient transfection

erk2 was cloned in pEGFP-C1 (Clontech, Mountain View, CA). Point mutations at the SPS motif (GFP-ERK2 EPE) were as previously described (Chuderland et al., 2008; Rubinfeld et al., 1999). Adding NLS on the C-terminus side of ERK2 was previously done in our lab. E14Tg2a cells were transfected with DNA constructs using Xfect reagent according to the manufacturer's instructions (TAKARA Bio, Japan). All siRNAs were from Dharmacon and were transfected using Dharmafect.

Coimmunoprecipitation

Cells were grown to 70% confluence and then serum starved (0.1% fetal bovine serum [FBS] for 16 h). After stimulation or other treatments, cells were rinsed twice with ice-cold phosphate-buffered saline (PBS) and once with buffer A. The cells were then scraped into buffer H (0.5 mL/plate), sonicated (60 W, 2 × 7 s), and centrifuged (15,000 rpm, 15 min). Supernatants were then incubated for 2 h (4°C, with rotation) with protein A/G-agarose beads (Santa Cruz Biotechnology) pre-linked to

specific Abs (1 h, 23°C). The bound protein A/G beads were washed three times with ice-cold coIP washing buffer. The immunoprecipitated beads were then resuspended with sample buffer and boiled; the resolved proteins were analyzed by western blotting with the indicated Abs.

Proximity ligation assay

Protein-protein interactions were detected by using a Duolink PLA kit (Olink Bioscience, Uppsala, Sweden), as previously described (Wortzel et al., 2021). Briefly, cells were grown, fixed, and permeabilized as described in the immunofluorescence staining section below. The samples were then incubated with Abs against two examined proteins (1 h, 23°C), washed (0.01 M Tris-HCl [pH 7.4], 0.15 M NaCl, and 0.05% Tween 20), and then incubated with specific probes (1 h, 37°C), followed by DAPI staining to visualize nuclei and then another wash (0.2 M Tris-HCl [pH 7.5], 0.15 M NaCl). The signal was visualized as distinct fluorescent spots by using spinning disk confocal microscopy. The number of PLA events was counted automatically in ImageJ, by the "analyze particles" feature, as previously described (Wortzel et al., 2015). Each field was counted twice: first, for the number of nuclei in the field, and second, for the number of PLA events. Then the average number of events was calculated per treatment. More than 100 cells were counted per treatment. Background correction, contrast adjustment, and quantification of the fluorescent signal were performed using Photoshop and ImageJ software.

Immunofluorescence staining

Cells were fixed in 4% paraformaldehyde in PBS (20 min, 23°C) and incubated with 2% bovine serum albumin (BSA) in PBS (15 min, 23°C), followed by permeabilization with Triton X-100 (0.1% in PBS, 5 min, 23°C). The fixed cells were then incubated with the primary Abs (1 h, 23°C), washed three times with PBS, and incubated with Alexa Fluor 488-conjugated, Alexa Fluor 568-conjugated, or Alexa Fluor 647-conjugated secondary Ab (1 h, 23°C) and DAPI. Slides were visualized by using spinning disk confocal microscopy (×63 magnification, Zeiss, Jena, Germany). Background correction and contrast adjustment of raw data images were performed using Photoshop (Adobe, San Jose, CA, USA)

Subcellular fractionation

Cells were grown to subconfluence and serum starved for 16 h. After treatments, the cells were rinsed twice with ice-cold PBS and once with ice-cold buffer A, scraped into buffer H, and then centrifuged (500*g*, 5 min at 4°C). The cells were resuspended with 600 μL buffer H, homogenized with 10 strokes of a 21G′ needle, 10 strokes of a 27G′ needle, and 50 strokes with a manual pestle. The homogenized cells were then centrifuged (5 min, 2,000*g*, 4°C) and homogenized again (same steps). The supernatant was the cytosolic fraction. The extract was washed twice with buffer H and once with buffer H + 0.1% NP-40 (centrifugation between washes at 2,800*g*, 5 min at 4°C). Then the extract was resuspended in 150 μL extraction buffer, kept on ice for 10 min, sonicated (60 W, 2 × 7 s), and centrifuged (15,000 rpm, 5 min at 4°C). The supernatant was the nuclear fraction.

Statistical analysis

Data are presented as means ± SE. The data were analyzed by a two-way ANOVA, except for Figure S2, which was done by three-way ANOVA, followed by *post hoc* Tukey or Dunnett's test. $p < 0.05$ was considered statistically significant.

Other methods

Other methods can be found in the supplemental information.

SUPPLEMENTAL INFORMATION

Supplemental information can be found online at https://doi.org/10.1016/j.stemcr.2022.11.017.

AUTHOR CONTRIBUTIONS

A.H.-L. designed and performed the experiments and wrote the article. R.S. supervised the study, designed the experiments, and wrote the article.

ACKNOWLEDGMENTS

We thank Drs. Jacob Hanna, Atan Gross, Denise Berti, and Galia Maik-Rachline and Ms. Tamar Hanoch for their help throughout the study. This study was supported by a grant from the Israel Science Foundation (ISF) to R.S. R.S. is an incumbent of the Yale Lewine and Ella Miller Lewine Professorial Chair for Cancer Research.

CONFLICT OF INTERESTS

The authors declare no competing interests.

Received: January 7, 2022
Revised: November 15, 2022
Accepted: November 19, 2022
Published: December 22, 2022

REFERENCES

Anjum, R., and Blenis, J. (2008). The RSK family of kinases: emerging roles in cellular signalling. Nat. Rev. Mol. Cell Biol. *9*, 747–758. nrm2509.

Azami, T., Bassalert, C., Allègre, N., Valverde Estrella, L., Pouchin, P., Ema, M., and Chazaud, C. (2019). Regulation of the ERK signalling pathway in the developing mouse blastocyst. Development *146*, dev177139. https://doi.org/10.1242/dev.177139.

Brons, I.G.M., Smithers, L.E., Trotter, M.W.B., Rugg-Gunn, P., Sun, B., Chuva de Sousa Lopes, S.M., Howlett, S.K., Clarkson, A., Ahrlund-Richter, L., Pedersen, R.A., and Vallier, L. (2007). Derivation of pluripotent epiblast stem cells from mammalian embryos. Nature *448*, 191–195.

Casar, B., Rodríguez, J., Gibor, G., Seger, R., and Crespo, P. (2012). Mxi2 sustains ERK1/2 phosphorylation in the nucleus by preventing ERK1/2 binding to phosphatases. Biochem. J. *441*, 571–578.

Chen, H., Guo, R., Zhang, Q., Guo, H., Yang, M., Wu, Z., Gao, S., Liu, L., and Chen, L. (2015). Erk signaling is indispensable for genomic stability and self-renewal of mouse embryonic stem cells. Proc. Natl. Acad. Sci. USA *112*, E5936–E5943.

Chuderland, D., Konson, A., and Seger, R. (2008). Identification and characterization of a general nuclear translocation signal in signaling proteins. Mol. Cell *31*, 850–861.

Deathridge, J., Antolović, V., Parsons, M., and Chubb, J.R. (2019). Live imaging of ERK signalling dynamics in differentiating mouse embryonic stem cells. Development *146*, dev172940. https://doi.org/10.1242/dev.172940.

Dhaliwal, N.K., Miri, K., Davidson, S., Tamim El Jarkass, H., and Mitchell, J.A. (2018). KLF4 nuclear export requires ERK activation and initiates exit from naive pluripotency. Stem Cell Rep. *10*, 1308–1323.

Dutta, D. (2013). Signaling pathways dictating pluripotency in embryonic stem cells. Int. J. Dev. Biol. *57*, 667–675.

Eblen, S.T. (2018). Extracellular-regulated kinases: signaling from Ras to ERK substrates to control biological outcomes. Adv. Cancer Res. *138*, 99–142.

Flores, K., Yadav, S.S., Katz, A.A., and Seger, R. (2019). The nuclear translocation of mitogen-activated protein kinases: molecular mechanisms and use as novel therapeutic target. Neuroendocrinology *108*, 121–131.

Formstecher, E., Ramos, J.W., Fauquet, M., Calderwood, D.A., Hsieh, J.C., Canton, B., Nguyen, X.T., Barnier, J.V., Camonis, J., Ginsberg, M.H., and Chneiweiss, H. (2001). PEA-15 mediates cytoplasmic sequestration of ERK MAP kinase. Dev. Cell *1*, 239–250.

Fukuda, M., Asano, S., Nakamura, T., Adachi, M., Yoshida, M., Yanagida, M., and Nishida, E. (1997). CRM1 is responsible for intracellular transport mediated by the nuclear export signal. Nature *390*, 308–311.

Hackett, J.A., and Surani, M.A. (2014). Regulatory principles of pluripotency: from the ground state up. Cell Stem Cell *15*, 416–430.

Hamilton, W.B., and Brickman, J.M. (2014). Erk signaling suppresses embryonic stem cell self-renewal to specify endoderm. Cell Rep. *9*, 2056–2070.

Hamilton, W.B., Mosesson, Y., Monteiro, R.S., Emdal, K.B., Knudsen, T.E., Francavilla, C., Barkai, N., Olsen, J.V., and Brickman, J.M. (2019). Dynamic lineage priming is driven via direct enhancer regulation by ERK. Nature *575*, 355–360.

Herrero, A., Pinto, A., Colón-Bolea, P., Casar, B., Jones, M., Agudo-Ibáñez, L., Vidal, R., Tenbaum, S.P., Nuciforo, P., Valdizán, E.M., et al. (2015). Small molecule inhibition of ERK dimerization prevents tumorigenesis by RAS-ERK pathway oncogenes. Cancer Cell *28*, 170–182.

Kholodenko, B.N., Hancock, J.F., and Kolch, W. (2010). Signalling ballet in space and time. Nat. Rev. Mol. Cell Biol. *11*, 414–426.

Kim, H., Wu, J., Ye, S., Tai, C.I., Zhou, X., Yan, H., Li, P., Pera, M., and Ying, Q.L. (2013). Modulation of beta-catenin function maintains mouse epiblast stem cell and human embryonic stem cell self-renewal. Nat. Commun. *4*, 2403. https://doi.org/10.1038/ncomms3403.

Kim, M.O., Kim, S.H., Cho, Y.Y., Nadas, J., Jeong, C.H., Yao, K., Kim, D.J., Yu, D.H., Keum, Y.S., Lee, K.Y., et al. (2012). ERK1 and ERK2 regulate embryonic stem cell self-renewal through phosphorylation of Klf4. Nat. Struct. Mol. Biol. *19*, 283–290.

Kim, S.H., Kim, M.O., Cho, Y.Y., Yao, K., Kim, D.J., Jeong, C.H., Yu, D.H., Bae, K.B., Cho, E.J., Jung, S.K., et al. (2014). ERK1 phosphorylates Nanog to regulate protein stability and stem cell self-renewal. Stem Cell Res. *13*, 1–11.

Kojima, Y., Kaufman-Francis, K., Studdert, J.B., Steiner, K.A., Power, M.D., Loebel, D.A.F., Jones, V., Hor, A., de Alencastro, G., Logan, G.J., et al. (2014). The transcriptional and functional properties of mouse epiblast stem cells resemble the anterior primitive streak. Cell Stem Cell *14*, 107–120.

Kraus, S., and Seger, R. (2004). Determination of ERK activity: anti-phospho-ERK antibodies, in vitro phosphorylation, and in-gel kinase assay. Methods Mol. Biol. *250*, 29–48.

Lavoie, H., Gagnon, J., and Therrien, M. (2020). ERK signalling: a master regulator of cell behaviour, life and fate. Nat. Rev. Mol. Cell Biol. *21*, 607–632.

Lee, S., Rauch, J., and Kolch, W. (2020). Targeting MAPK signaling in cancer: mechanisms of drug resistance and sensitivity. Int. J. Mol. Sci. *21*, E1102. https://doi.org/10.3390/ijms21031102.

Ma, X., Chen, H., and Chen, L. (2016). A dual role of Erk signaling in embryonic stem cells. Exp. Hematol. *44*, 151–156.

Maik-Rachline, G., Hacohen-Lev-Ran, A., and Seger, R. (2019). Nuclear ERK: mechanism of translocation, substrates, and role in cancer. Int. J. Mol. Sci. *20*, 1194. https://doi.org/10.3390/ijms20051194.

Maik-Rachline, G., Zehorai, E., Hanoch, T., Blenis, J., and Seger, R. (2018). The nuclear translocation of the kinases p38 and JNK promotes inflammation-induced cancer. Sci. Signal. *11*, eaao3428. https://doi.org/10.1126/scisignal.aao3428.

Meng, Y., Moore, R., Tao, W., Smith, E.R., Tse, J.D., Caslini, C., and Xu, X.X. (2018). GATA6 phosphorylation by Erk1/2 propels exit from pluripotency and commitment to primitive endoderm. Dev. Biol. *436*, 55–65.

Michailovici, I., Harrington, H.A., Azogui, H.H., Yahalom-Ronen, Y., Plotnikov, A., Ching, S., Stumpf, M.P.H., Klein, O.D., Seger, R., and Tzahor, E. (2014). Nuclear to cytoplasmic shuttling of ERK promotes differentiation of muscle stem/progenitor cells. Development *141*, 2611–2620.

Morey, L., Santanach, A., and Di Croce, L. (2015). Pluripotency and epigenetic factors in mouse embryonic stem cell fate regulation. Mol. Cell Biol. *35*, 2716–2728.

Mossahebi-Mohammadi, M., Quan, M., Zhang, J.S., and Li, X. (2020). FGF signaling pathway: a key regulator of stem cell pluripotency. Front. Cell Dev. Biol. *8*, 79. https://doi.org/10.3389/fcell.2020.00079.

Ochiai, H., Hayashi, T., Umeda, M., Yoshimura, M., Harada, A., Shimizu, Y., Nakano, K., Saitoh, N., Liu, Z., Yamamoto, T., et al. (2020). Genome-wide kinetic properties of transcriptional bursting in mouse embryonic stem cells. Sci. Adv. *6*, eaaz6699. https://doi.org/10.1126/sciadv.aaz6699.

Papatsenko, D., Darr, H., Kulakovskiy, I.V., Waghray, A., Makeev, V.J., MacArthur, B.D., and Lemischka, I.R. (2015). Single-cell Analyses of ESCs reveal alternative pluripotent cell states and molecular mechanisms that control self-renewal. Stem Cell Rep. *5*, 207–220. https://doi.org/10.1016/j.stemcr.2015.07.004.

Plotnikov, A., Chuderland, D., Karamansha, Y., Livnah, O., and Seger, R. (2019). Nuclear ERK translocation is mediated by protein kinase CK2 and accelerated by autophosphorylation. Cell. Physiol. Biochem. *53*, 366–387.

Plotnikov, A., Flores, K., Maik-Rachline, G., Zehorai, E., Kapri-Pardes, E., Berti, D.A., Hanoch, T., Besser, M.J., and Seger, R. (2015). The nuclear translocation of ERK1/2 as an anticancer target. Nat. Commun. *6*, 6685. https://doi.org/10.1038/ncomms7685.

Rais, Y., Zviran, A., Geula, S., Gafni, O., Chomsky, E., Viukov, S., Mansour, A.A., Caspi, I., Krupalnik, V., Zerbib, M., et al. (2013). Deterministic direct reprogramming of somatic cells to pluripotency. Nature *502*, 65–70.

Rubinfeld, H., Hanoch, T., and Seger, R. (1999). Identification of a cytoplasmic-retention sequence in ERK2. J. Biol. Chem. *274*, 30349–30352.

Sangel, P., Oka, M., and Yoneda, Y. (2014). The role of Importin-betas in the maintenance and lineage commitment of mouse embryonic stem cells. FEBS Open Bio. *4*, 112–120.

Schevzov, G., Kee, A.J., Wang, B., Sequeira, V.B., Hook, J., Coombes, J.D., Lucas, C.A., Stehn, J.R., Musgrove, E.A., Cretu, A., et al. (2015). Regulation of cell proliferation by ERK and signal-dependent nuclear translocation of ERK is dependent on Tm5NM1-containing actin filaments. Mol. Biol. Cell *26*, 2475–2490. https://doi.org/10.1091/mbc.E14-10-1453.

Shaul, Y.D., and Seger, R. (2007). The MEK/ERK cascade: from signaling specificity to diverse functions. Biochim. Biophys. Acta *1773*, 1213–1226.

Watson, U., Jain, R., Asthana, S., and Saini, D.K. (2018). Spatiotemporal modulation of ERK activation by GPCRs. Int. Rev. Cell Mol. Biol. *338*, 111–140.

Weinberger, L., Ayyash, M., Novershtern, N., and Hanna, J.H. (2016). Dynamic stem cell states: naive to primed pluripotency in rodents and humans. Nat. Rev. Mol. Cell Biol. *17*, 155–169.

Wortzel, I., Hanoch, T., Porat, Z., Hausser, A., and Seger, R. (2015). Mitotic Golgi translocation of ERK1c is mediated by a PI4KIIIbeta-14-3-3gamma shuttling complex. J. Cell Sci. *128*, 4083–4095.

Wortzel, I., Maik-Rachline, G., Yadav, S.S., Hanoch, T., and Seger, R. (2021). Mitotic HOOK3 phosphorylation by ERK1c drives microtubule-dependent Golgi destabilization and fragmentation. iScience *24*, 102670. https://doi.org/10.1016/j.isci.2021.102670.

Wortzel, I., and Seger, R. (2011). The ERK cascade: distinct functions within various subcellular organelles. Genes Cancer *2*, 195–209. https://doi.org/10.1177/1947601911407328.

Ying, Q.L., Nichols, J., Chambers, I., and Smith, A. (2003). BMP induction of Id proteins suppresses differentiation and sustains embryonic stem cell self-renewal in collaboration with STAT3. Cell *115*, 281–292.

Ying, Q.L., Wray, J., Nichols, J., Batlle-Morera, L., Doble, B., Woodgett, J., Cohen, P., and Smith, A. (2008). The ground state of embryonic stem cell self-renewal. Nature *453*, 519–523.

Zehorai, E., and Seger, R. (2019). Beta-like importins mediate the nuclear translocation of MAPKs. Cell. Physiol. Biochem. *52*, 802–821.

Stem Cell Reports, Volume *18*

Supplemental Information

Retention of ERK in the cytoplasm mediates the pluripotency of embryonic stem cells

Avital Hacohen Lev-Ran and Rony Seger

Retention of ERK in the cytoplasm mediates the pluripotency of embryonic stem cells

Supplementary Figures

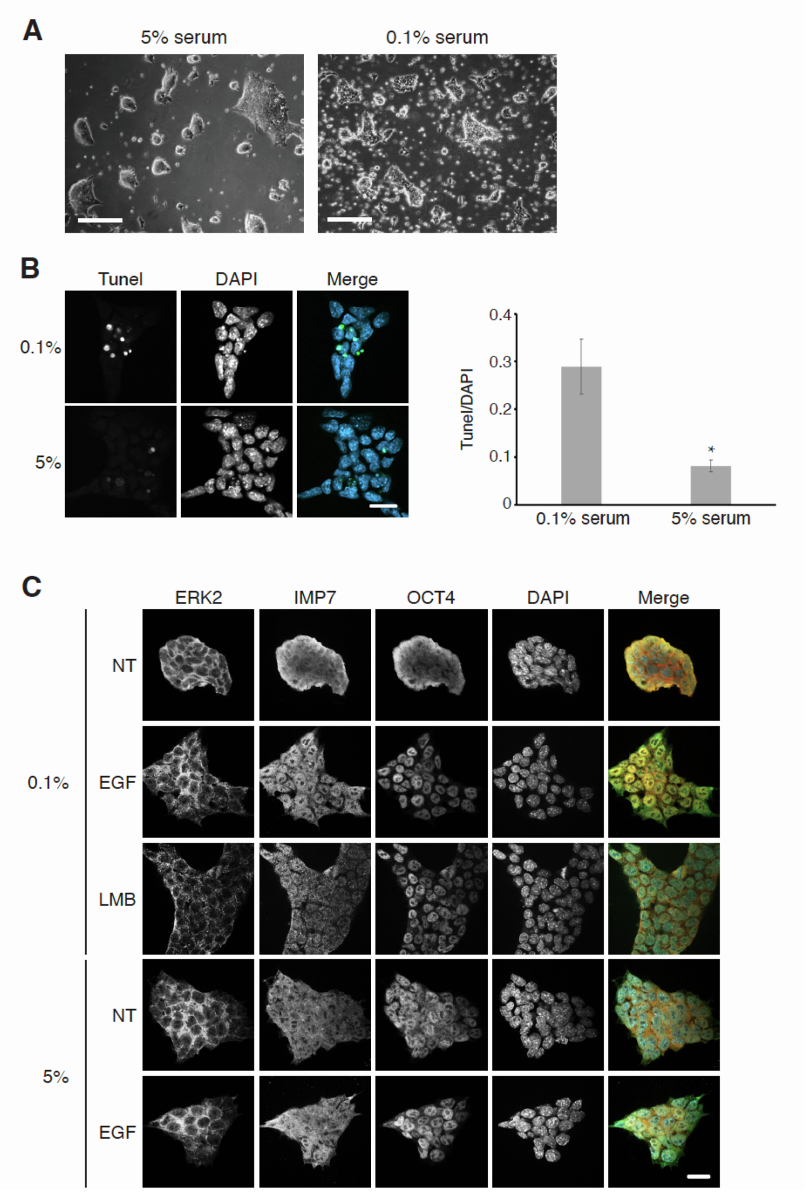

Figure S1. The effect of different starvation medium on mESC, related to Experimental Procedures. **(A)** Morphological studies. E14Tg2a cells were grown in two different low serum concentrations: 0.1% and 5% for 16 hr and the following parameters were followed. The morphology of colonies was visualized by a regular light microscope. Scale bars - 500µm. (**B**) TUNEL assay. The fluorescence was visualized by spinning disk confocal microscopy. Scale bar – 20 µm. The data presented in the bar-graph in the right side represents means ± SE of 3 experiments. *$P < 0.05$ as calculated by T test. (**C**) Effect of 0.1% serum on stimulated ERK translocation compared to cells starved in 5% FCS. The serum-starved (either 0.1% or 5%) cells were treated with EGF (50 ng/ml, 15 min), or left untreated, (NT). The cells starved in 0.1% FCS were also stimulated with LMB (5 ng/ml, 1 hr). Then, the cells were fixed and stained with the indicated Abs. The nuclei were detected using DAPI. Fluorescence was visualized by spinning disk confocal microscopy. Scale bar – 20 µm.

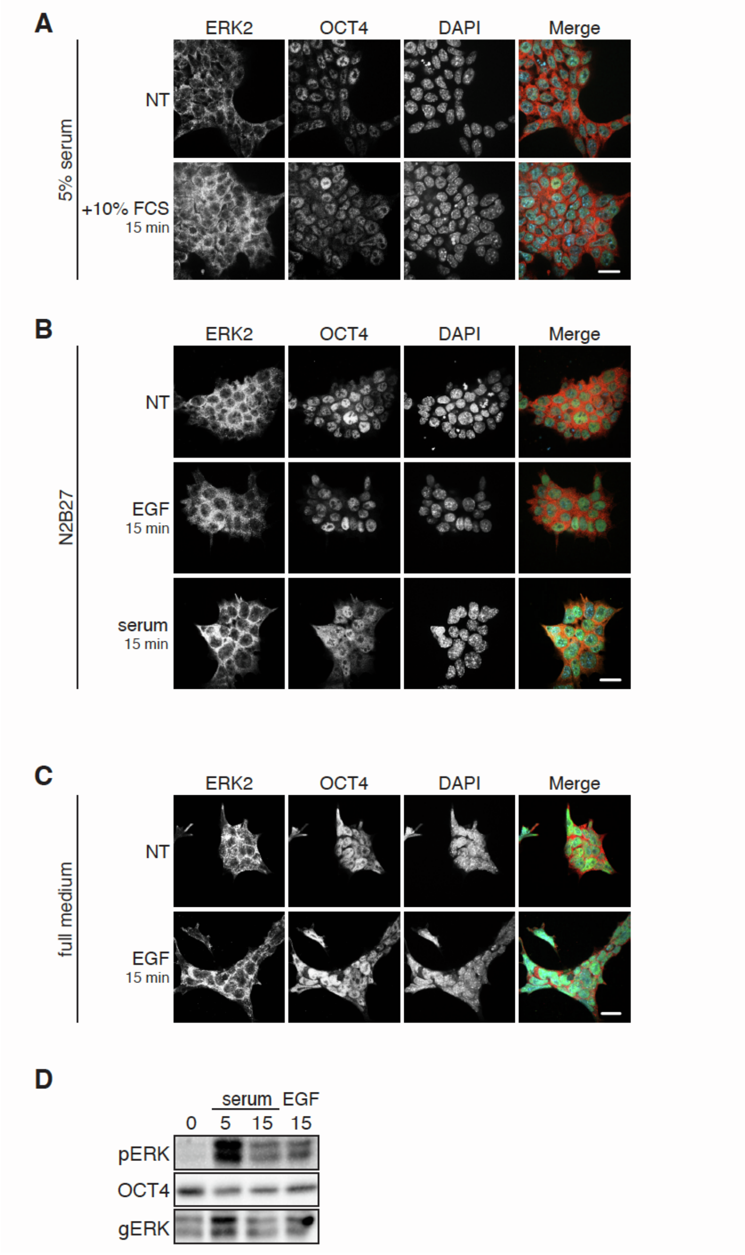

Figure S2. The effect of serum/ serum free medium on ERK translocation in mESC, related to Fig. 1 and Experimental Procedures). (**A**) Lack of nuclear ERK translocation upon serum stimulation. Fluorescence micrographs of ERK2 and OCT4 in E14Tg2a cells grown in reduced serum concentrations (5%, 16 hr) and stimulated with 10% FCS (total 15% serum, 15 min) or left untreated. Scale bar - 20 µm in all. (**B**) Lack of stimulated nuclear translocation of ERK grown in serum-free medium. Fluorescence micrographs of ERK2 and OCT4 in E14Tg2a cells grown in N2B27 (2 days), and then stimulated with EGF (50 ng/ml, 15 min), FCS (15% serum, 15 min) or left untreated as control (NT). (**C**) Lack of stimulated ERK translocation without starvation. Fluorescence micrographs of ERK2 and OCT4 in E14Tg2a cells grown in full medium (15% serum, 2 days) and stimulated with EGF (50 ng/ml, 15 min), or left untreated as control (NT). Fluorescence was visualized by spinning disk confocal microscopy. (D) ERK is activated by EGF and serum in E14Tg2a cells grown without serum. The cells were grown in N2B27 (2 days) and then were either stimulated with EGF (50 ng/ml, 15 min), FCS (15% serum, 5 and 15 min) or left untreated (0). The cells were then harvested and subjected to Western blotting using the indicated Abs.

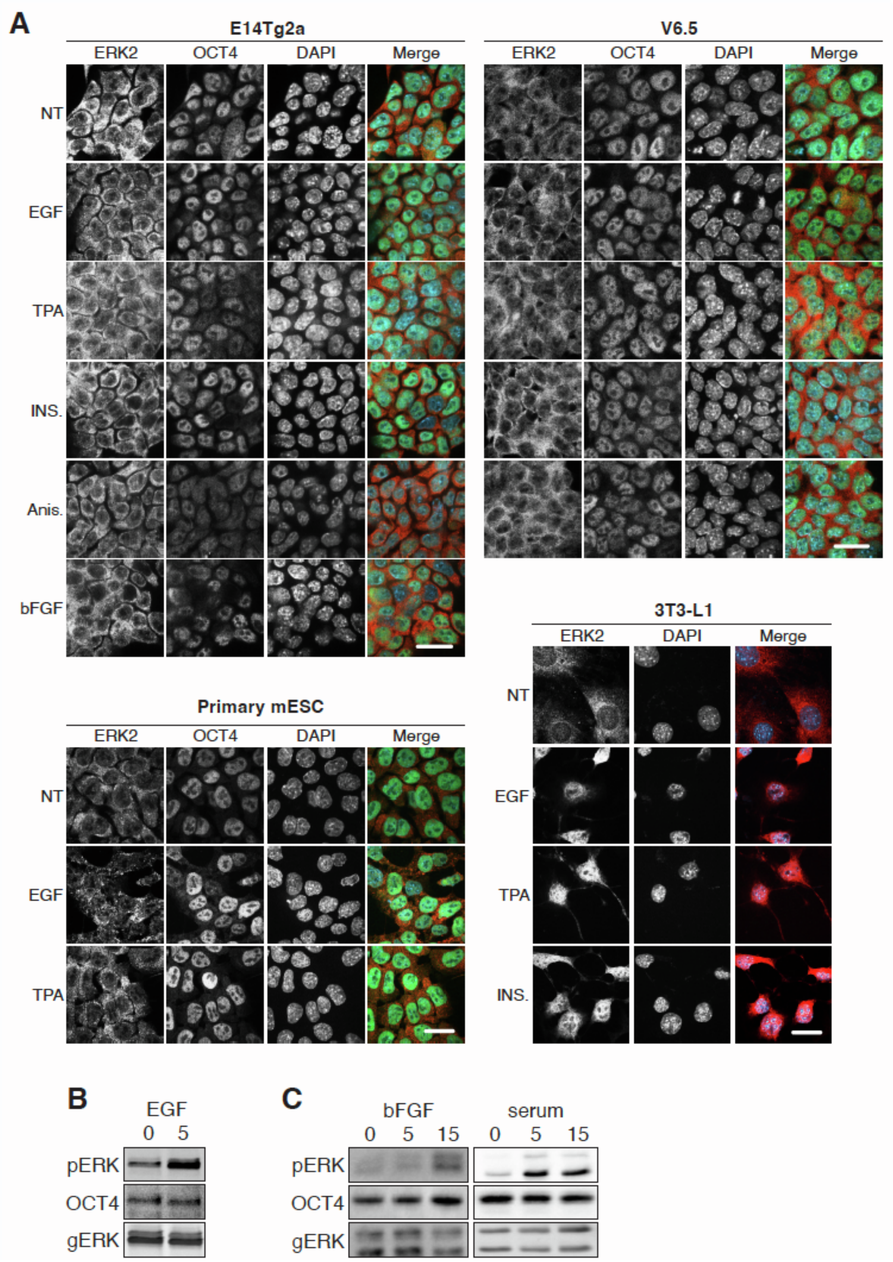

Figure S3. ERK1/2 localization in different cells and stimuli, related to Fig. 1. (**A**) Lack of nuclear translocation in various stem cells. Fluorescence micrographs of ERK2 and OCT4 in E14Tg2a, V6.5, primary (blastocyst-derived) stem cells and 3T3-L1 cells. The indicated cells were grown with reduced serum concentrations for 16 hr (mESC - 5%; 3T3-L1 – 0.1%) and then were stimulated with EGF (50 ng/ml), TPA (250 nM), insulin (100 nM; INS.), anisomycin (1 μg/ml; Anis.) and bFGF (50 ng/ml) for 15 min or left untreated (NT) as indicated. The nuclei were detected using DAPI. Fluorescence was visualized by spinning disk confocal microscopy. Scale bars - 20 μm in all. (**B**) ERK is activated by EGF in primary stem cells. The cells were grown in reduced serum concentration (5%, 16 hr) as above, and then were either stimulated with EGF (50 ng/ml, 5 min) or left untreated (0). The cells were then harvested and subjected to Western blotting using the indicated Abs. (**C**) ERK is activated by bFGF and Serum. E14Tg2a cells were grown in reduced serum concentration (5%, 16 hr) as above, and then were either stimulated with either bFGF (50 ng/ml, 5 or 15 min), 10% FCS (total 15% serum, 5 or 15 min) or left untreated (0). The cells were then harvested and subjected to Western blotting using the indicated Abs.

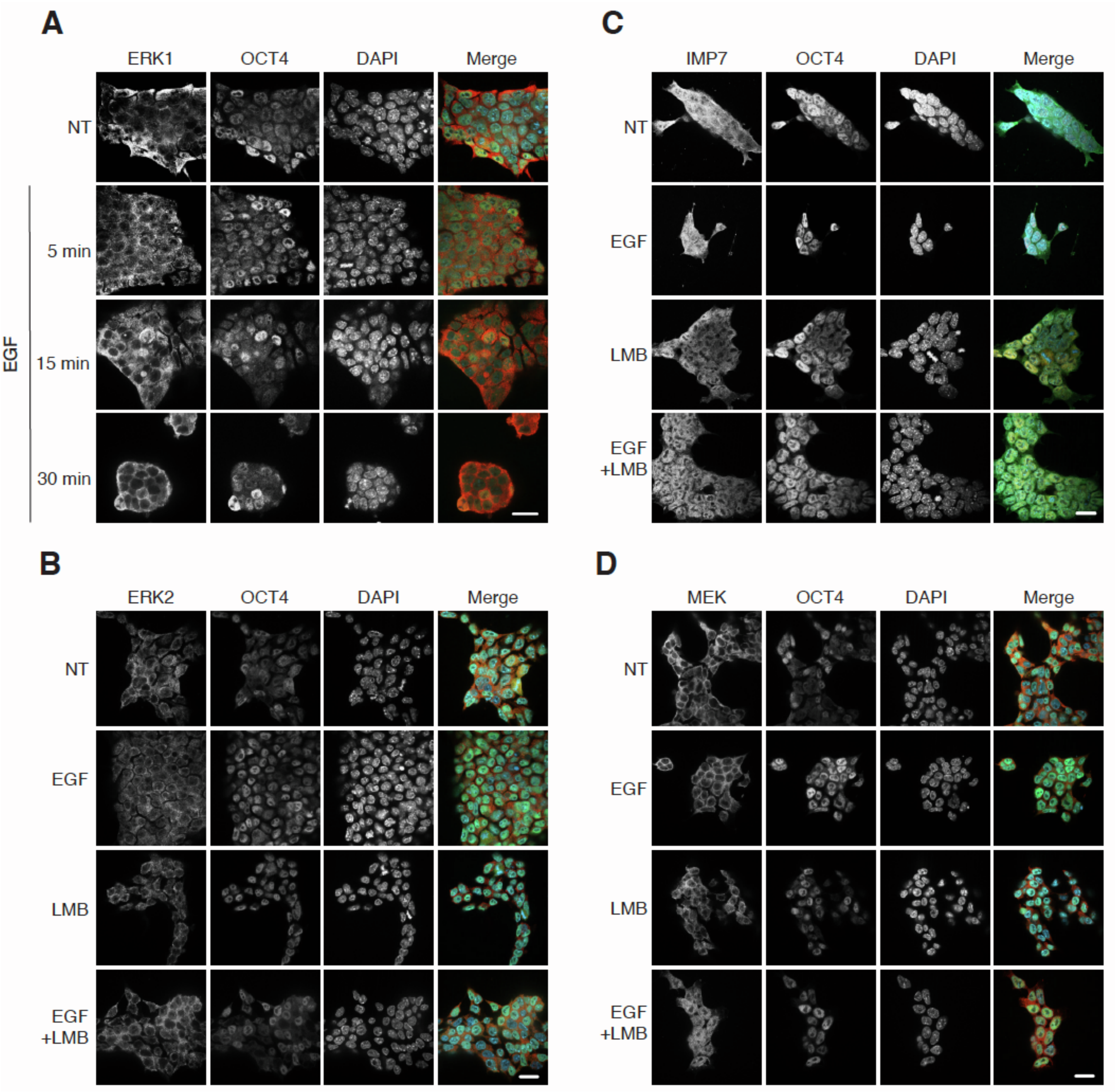

Figure S4. Subcellular localization of ERK1+2, IMP7 and MEK upon various stimulation of mESC, related to Fig. 1. (A) Subcellular localization of ERK1 upon EGF stimulation in different time points. Fluorescence micrographs of ERK1 (using specific Ab) in E14Tg2a cells grown in reduced serum concentrations (5%, 16 hr) and then either stimulation with EGF (50 ng/ml, 5, 15 and 30 min) or no stimulation (NT). Next the cells were fixed and stained with the indicated Abs. The nuclei were detected using DAPI. Fluorescence was visualized by spinning disk confocal microscopy. Scale bars - 20 µm in all. (**B-D**) Leptomycin B does not affect ERK2 in mESC. E14Tg2a cells were grown in low serum concentrations (5%, 16 hr) and then treated with EGF (50 ng/ml, 15 min), Leptomycin B (LMB, 5 ng/ml, 1 hr), LMB together with EGF that was added for the last 15 min of LMB treatment (total 1 hr) or left untreated as control (NT). Then the cells were fixed and stained with the ERK (B), IMP7 (C) or MEK (D) Abs with anti OCT4 Ab and DAPI. Fluorescence was visualized by spinning disk confocal microscopy.

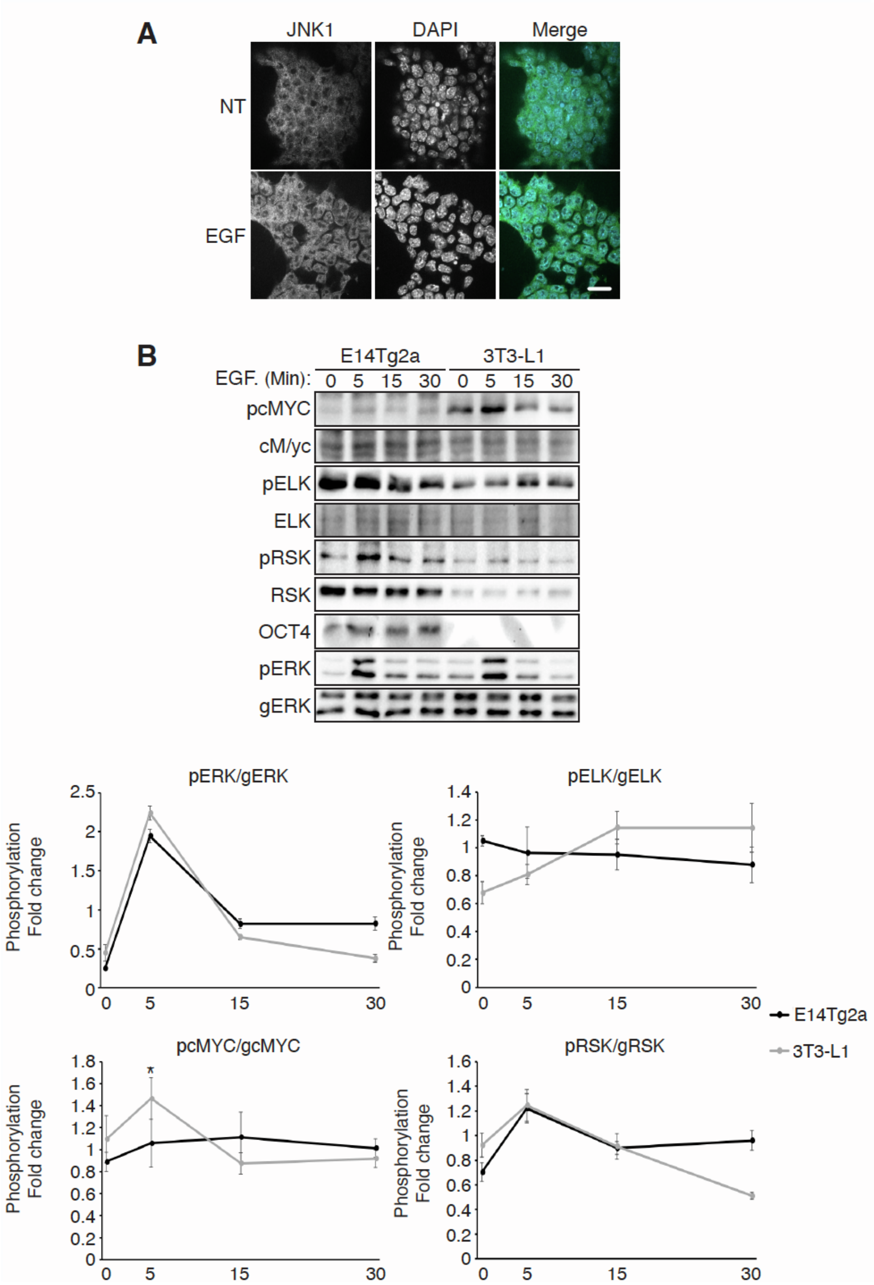

Figure S5: Studies on JNK1 and ERK substrates localization or activation upon EGF treatment, related to Fig. 2. (A) Nuclear translocation of JNK1 upon EGF stimulation of mESC. E14Tg2a cells were grown in low serum concentrations (5%, 16 hr) and then treated with EGF (50 ng/ml, 15 min), or left untreated as control (NT). Then the cells were fixed and stained with anti JNK1 Ab and DAPI. Fluorescence was visualized by spinning disk confocal microscopy. Scale bar - 20 µm. **(B)** Phosphorylation of cytoplasmic but not nuclear ERK1/2 substrates. E14Tg2a and 3T3-L1 cells grown in reduced serum concentration (E14Tg2 - 5%; 3T3-L1 – 0.1%) for 16 hr and either stimulated (E14Tg2a cells with EGF (50 ng/ml) and 3T3-L1 with insulin (100 nM)) for 5, 15 or 30 min or left untreated (0). The cells were then harvested and subjected to western blot analysis with the indicated Abs. The quantification in the graph below represent means ± SE of three experiments. **P* <0.05 by Tukey test, indicate significant changes between stem cells line (E14Tg2a) and the control cells (3T3-L1).

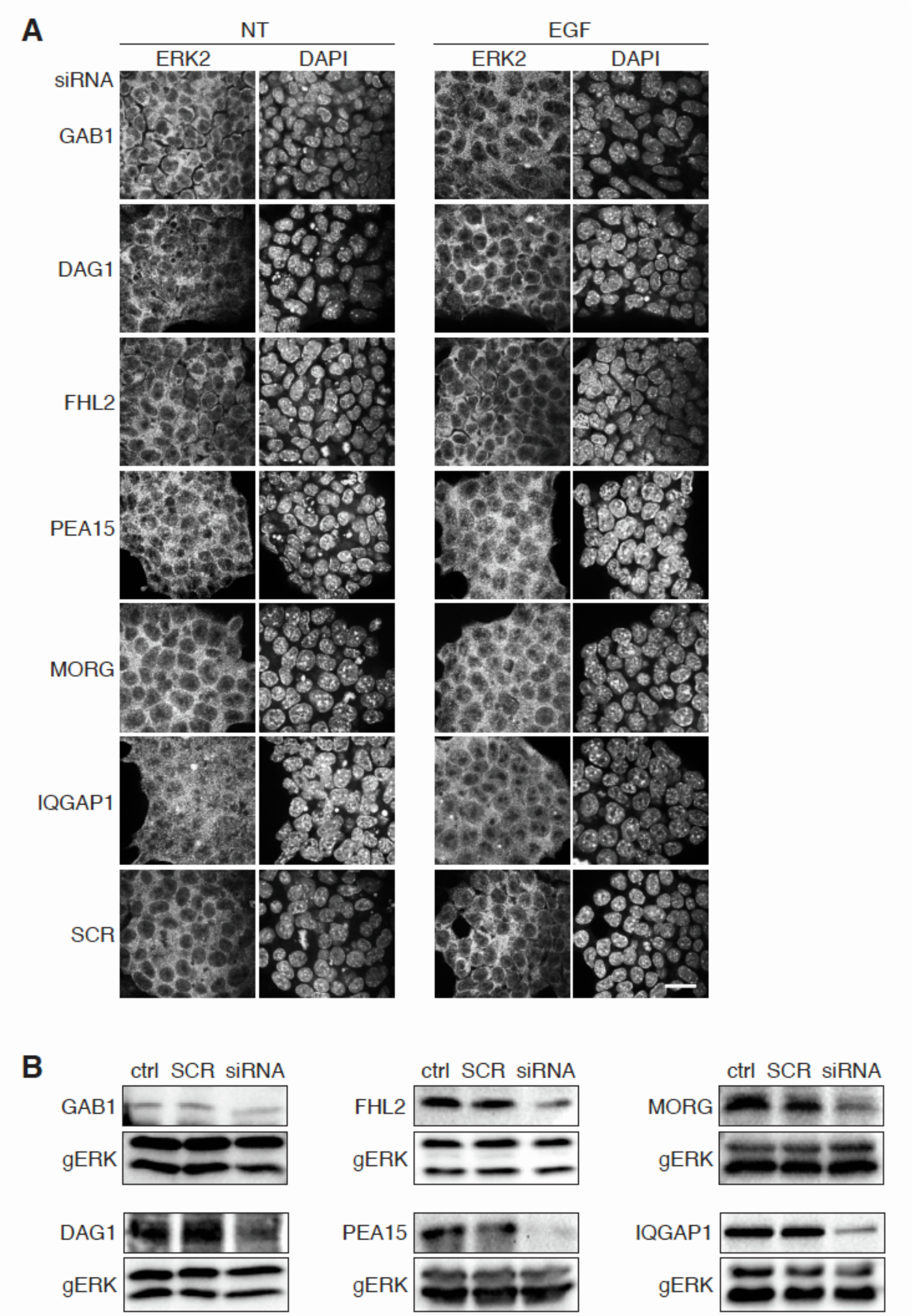

Figure S6: The effect of reduced expression of anchoring proteins on ERK1/2 localization, related to Fig. 3. (A) ERK localization. E14Tg2a cells were treated with 100nM of siRNA against GAB1, DAG1, FHL2, PEA15, MORG, IQGAP1 or scrambled (SCR) siRNA, then grown in low serum concentrations (5%, 16 hr) and the either stimulated with EGF (50 ng/ml, 15 min) or left untreated (NT). Next, the cells were fixed and stained with the indicated Abs. The fluorescence was visualized by spinning disk confocal microscopy. Scale bar - 20 µm. **(B)** Western blot analysis. The cells were treated as above, followed by extraction and Western blotting with the indicated Abs.

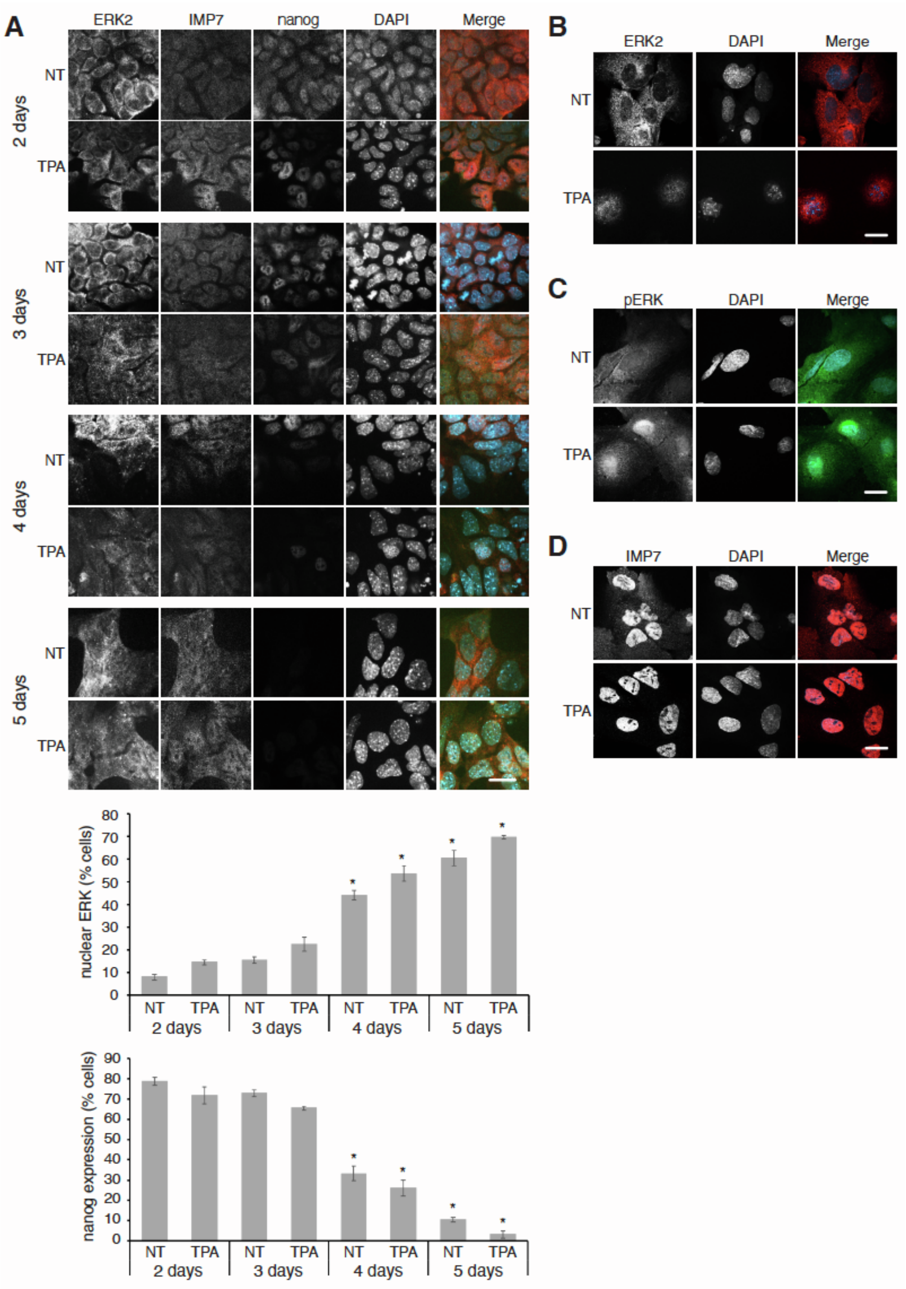

Figure S7: ERK localization during naïve to primed transition and in EpiSC, related to Fig. 5. (A) Expansion of figure 5. E14Tg2a cells were subjected to naïve to primed transition as in Fig. 5. In days 2, 3, 4 and 5 of the transition, some of the cells were stimulated with TPA (250 nM, 15 min), or left untreated (NT). The cells were then fixed and stained with the indicated Abs. Fluorescence was visualized by spinning disk confocal microscopy. The bar-graphs below represent percentage of cells that have nuclear ERK (left) and that express nanog (right). Means ± SE of three experiments. $*P < 0.01$ by paired T test indicates a significant change as compared with day 2. Scale bars - 20 µm in all **(B-D)** Nuclear translocation of ERK and IMP7 in EpiSCs. EpiSCs were produced from E14Tg2A for 6 passages. Fluorescence micrographs of ERK2 (B), pERK (C) and IMP7 (D) in these cells that were stimulated with TPA (250 nM 15 min) or left untreated (NT). Fluorescence was visualized by spinning disk confocal microscopy.

Supplemental Experimental Procedures

Reagents and antibodies. Tetradecanoyl phorbol acetate (TPA), epidermal growth factor (EGF), basic fibroblast growth factor (bFGF), anisomycin, CHIR99021, PD0325901, leptomycin B, insulin and 4,6-diamino-2-phenylindole (DAPI) were purchased from Sigma-Aldrich (Rehovot, Israel). FGF2 (100-18C) and Activin A (120-14) were from Peprotech (Rehovot Israel). Protein A/G PLUS-agarose beads were obtained from Santa Cruz Biotechnology, Inc. (CA, USA). Bovine serum albumin (BSA) was purchased from MP biomedical (OH, USA). ECL was from Biological Industries (Beit-Haemek, Israel). Lipofectamine2000 was from Thermo Fisher Scientific, (MA, USA). Recombinant human BMP4 from R&D Systems (Mn, USA). Anti-general (g) ERK1 (C-16) Cat #sc-93, anti-gERK2 (C-14) Cat #sc-154, anti-OCT3/4 Cat #sc-5279, anti-nanog Cat #sc-3769, anti-IMP7 Cat #sc-365231+ #sc-134913, anti-Elk1 Cat #sc-355, anti-pElk1 Cat #sc-8406, anti-RSK1 Cat #sc-231 , anti-JNK1/3 (C-17) Cat # sc-474, anti-IQGAP1 Cat #sc-376021 and normal IgG Cat #sc-68786 Abs were obtained from Santa Cruz Biotechnology (CA, USA). Anti-IMP7 Ab Cat #H00010527-M07 was obtained from Abnova (Taipei, Taiwan). Anti-pERK1/2 (p(TEY)ERK1/2) Cat #M8159, anti-gERK1/2 Cat #M5670, anti-gERK1 Cat #M7927 and anti-gMEK Cat #M5795 Abs were obtained from Sigma-Aldrich (Rehovot, Israel). Anti-Histone3 Cat #ab1791, anti-phospho cMyc Cat #ab51156 and anti-IMP7 Cat #ab15840 Abs were obtained from Abcam (Cambridge, UK). Anti-cMyc Cat #5605 and anti-pRSK (p90) Cat #9346S Abs were obtained from Cell Signaling (MA, USA) Anti-phospho SPS-ERK1/2 (1:500) Abs were produced in Biological Service Unit of the Weizmann Institute of Science (Rehovot, Israel). Secondary fluorescent Ab Cat #A10042, #A11008 were from Molecular Probes (Eugene, OR). Secondary Abs conjugated to horseradish peroxidase (HRP) Cat #111-035-003, #115-035-003 were obtained from Jackson ImmunoResearch laboratories (PA, USA)

The two peptides used were EPE - GQLNHILGILGEPEQED and Scramble— GNILSQELPHSGDLQIG (Plotnikov et al., 2015). Each of them was conjugated in its N-terminal to myristic acid and C-terminal amidated. The peptides were purchased from Gnenscript (NJ, USA). They were >85% pure and kept as 100 mM DMSO stock solution at −20 °C (Plotnikov *et al.*, 2015).

Buffers.

Buffer A - 50 mM β-glycerophosphate pH 7.3, 1.5 mM EGTA, 1 mM EDTA, 1mM dithiothreitol, and 0.1 mM sodium vanadate.

Buffer H – The same ingredients as Buffer A plus 0.1 mM sodium vanadate, 10 μg/ml aprotinin, 10 μg/ml leupeptin, 2 μg/ml pepstatin A, and 1 mM Benzamidin.

Radio-immunoprecipitation assay (RIPA) buffer - 137 mM NaCl, 20 mM Tris (pH 7.4), 10% glycerol, 1% Triton X-100, 0.5% deoxycholate, 0.1% SDS, 2 mM EDTA, 1 mM phenylmethylsulfonyl flouride (PMSF) and 20 μM leupeptin.

Co-IP Washing buffer – 20 mM HEPES pH 8.0, 2 mM $MgCl_2$, 2 mM EGTA, 100 mM NaCl.

Extraction buffer for the fractionation assay– 4 M NaCl, 1 M β-glycerophosphate, 0.1M Na_3VO_4, 0.2 M $MgCl_2$, 0.5 M EDTA, 1 M DTT, 25% glycerol.

Cell cultures.

The mESC lines E14Tg2a and V6.5 (Beard *et al*., 2006), were grown on plates coated with 0.2% gelatin in Glasgow Minimum Essential Medium (GMEM) supplemented with 15% fetal calf serum (FCS; Hyclone, ThermoFisher), 50 U/ml penicillin and streptomycin, 50 μg/ml glutamine, 1 mM Na Pyruvate, 0.1 mM β-mercaptoethanol, 1% MEM-Non-Essential Amino Acids (ThermoFisher) and 1000 U/ml Leukemia Inhibitory Factor (LIF; Merck, USA). For maintenance, V6.5 were cultured with irradiated mouse embryonic fibroblasts (MEFs; feeders) that were previously seeded on 0.1% gelatin. Primary (blastocyst-derived) mouse embryonic stem cells were produced with help from the Gross lab at the Weizmann Institute of Science (approved by the institutional Animal Care and Use Committee to the Gross lab). The cells were taken from pregnant mice, at 3.5 days post fertilization. The blastocysts were taken from the uterine horns, washed three times with the growing medium, and seeded on MEFs in a growing medium for a few days, until a mass of cells was formed, which are the embryonic stem cells. For maintenance, the cells were cultured with feeders and were grown in the same medium as E14Tg2a and V6.5 cells. ΔPE-OCT4-GFP transgenic reporter for pluripotency state of the mouse embryonic stem cells (Rais et al., 2013) were received from the Hanna's lab, Weizmann Institute of Science. The cells were grown in same condition as V6.5 cells. Their GFP levels were measured by FACS. 3T3-L1 cells were cultured in Dulbecco's modified Eagle's medium (DMEM) supplemented with 50 U/ml penicillin and streptomycin, 50 μg/ml glutamine and 10% fetal calf serum (FCS).

For naïve to primed and peptides experiments the cells were grown on plates coated growth factor reduced Matrigel (Corning, USA), in N2B27 medium contained DMEM-F12 and neurobasal at a 1:1 ratio supplemented with 0.5% N2 supplement (Gibco), 1% B27 supplement (Gibco), 50 U/ml penicillin and streptomycin, 50 μg/ml glutamine, 1 mM Na Pyruvate, 0.1mM β-

mercaptoethanol, 1% MEM Non-Essential Amino Acids All these reagents were from Gibco ThermoFisher. For naïve to primed transition, N2B27 media contained 12 ng/ml recombinant human FGF2 (Peprotech, Rehovot Israel) and 20 ng/ml recombinant human activin-A. In the peptides experiment, 3 µM CHIR99021 and recombinant mouse LIF 1000 U/ml were added to all conditions. PD0325901 1 µM, DMSO (1 µl/ml) or peptides 10 µM were added to each treatment. For serum free experiment, N2B27 media contained Recombinant human BMP4 (100 µg/ml) and recombinant mouse LIF 1000 U/ml for 2 passages. Mouse Embryonic Fibroblast (MEF) cells were grown on plates coated 0.2% gelatin in DMEM supplemented 50 U/ml penicillin and streptomycin, 50 µg/ml glutamine, 1 mM Na Pyruvate and 10% fetal calf serum (FCS). The "feeders" endow trophic factors that together with fetal calf serum and LIF support self-renewal. All cells were maintained at 37°C in a humidified atmosphere of 95% air and 5% CO_2.

EpiSCs were produced form E14Tg2A following the protocol published in reference (Brons et al., 2007). The cells were grown 2 passages with 2i+LIF media (N2B27 with 1 µM PD0325901, 3 µM CHIR99021 and LIF 1000 U/ml) and then transformed to N2B27 contained 1% FCS, FGF2 (12 ng/ml) and Activin A (20 ng/ml) for 4 passages. The cells were grown on plates coated growth factor reduced Matrigel for the whole procedure.

MEFs were prepared under sterile conditions. The pregnant female mouse (13.5 d.p.c.) was sacrificed. The uterine horns were dissected out and placed into a petri dish containing PBS. Each embryo was separated from its placenta and surrounding membranes and head, liver and gut were removed. The embryos were washed by transferred them to a petri dish containing clean PBS few times (to remove as much red blood cells as possible). Each embryo was homogenized with 1 ml medium by using 1cc syringe with an 18G 11/2" needle 7 times up and down. The suspension was plated on gelatinized plates, 1-1.5 embryos /10 cm plate in MEF medium. After four passages the cells were irradiated at 3000 rads. These cells were used as feeder for culturing primary mESCs.

Preparation of cell extracts and Western blotting. Cells were grown to subconfluence and serum-starved for 16 hr. After treatments, the cells were rinsed twice with ice-cold PBS and once with ice-cold Buffer A, scraped into Buffer H and disrupted by sonication (60 W, 2 × 7 sec) on ice or scraped into RIPA buffer. The extracts were centrifuged (15,000 rpm, 15 min at 4° C) and the supernatants were either subjected to co-immunoprecipitation or resuspended and boiled for 5 min in sample buffer. The samples were then subjected to 12% or 10% SDS-PAGE and Western blotting with the appropriate Abs, which were detected using alkaline phosphatase

or ECL according to the manufacturer's instructions. A representative Western blot of at least three independent experiments is shown for all cases.

TUNEL assay: in situ cell death was detected by using a TUNEL [terminal deoxynucleotidyl transferase (TdT)] kit (Roche Molecular Biochemicals). Cells were fixed in 4% paraformaldehyde in PBS (20 min, 23° C) and incubated with 2% BSA in PBS (15 min, 23°C), followed by permeabilization with Triton X-100 (0.1% in PBS, 5 min, 23° C). The fixed cells were washed again with PBS and incubated with terminal deoxynucleotidyltransferase-mediated nick end labeling (TUNEL) reaction mixture containing fluorescein-dUTP and terminal deoxynucleotidyl transferase for 30 min at 37° C. Preparations were analyzed by fluorescence microscopy. TUNEL staining of cells per total cell number was calculated. More than 100 cells were counted per treatment.

References

Beard, C., Hochedlinger, K., Plath, K., Wutz, A., and Jaenisch, R. (2006). Efficient method to generate single-copy transgenic mice by site-specific integration in embryonic stem cells. Genesis *44*, 23-28. 10.1002/gene.20180.

Brons, I.G., Smithers, L.E., Trotter, M.W., Rugg-Gunn, P., Sun, B., Chuva de Sousa Lopes, S.M., Howlett, S.K., Clarkson, A., Ahrlund-Richter, L., Pedersen, R.A., and Vallier, L. (2007). Derivation of pluripotent epiblast stem cells from mammalian embryos. Nature *448*, 191-195. 10.1038/nature05950.

Plotnikov, A., Flores, K., Maik-Rachline, G., Zehorai, E., Kapri-Pardes, E., Berti, D.A., Hanoch, T., Besser, M.J., and Seger, R. (2015). The nuclear translocation of ERK1/2 as an anticancer target. Nat Commun *6*, 6685. 10.1038/ncomms7685.

Rais, Y., Zviran, A., Geula, S., Gafni, O., Chomsky, E., Viukov, S., Mansour, A.A., Caspi, I., Krupalnik, V., Zerbib, M., et al. (2013). Deterministic direct reprogramming of somatic cells to pluripotency. Nature *502*, 65-70. 10.1038/nature12587.
